# Next-generation bio-packaging systems for food safety and shelf-life extension: controlled-release antimicrobial strategies and migration control

**DOI:** 10.3389/fnut.2026.1948313

**Published:** 2026-09-24

**Authors:** Lutfun Nahar, Yumei Zhang, Jinping Li, Daijie Wang, Satyajit D. Sarker

**Affiliations:** 1 Laboratory of Growth Regulators, Palacký University and Institute of Experimental Botany, The Czech Academy of Sciences, Olomouc, Czechia; 2 Centre for Natural Products Discovery, School of Pharmacy and Biomolecular Sciences, Liverpool John Moores University, Liverpool, United Kingdom; 3 College of Agronomy, Qingdao Agricultural University, Chengyang, Qingdao, China; 4 Research Center of SCO Countries, Qilu University of Technology, Jinan, China; 5 International Joint Laboratory of Medicinal Food Development and Health Products Creation, Biological Engineering Technology Innovation Center of Shandong Province, Heze Branch of Qilu University of Technology (Shandong Academy of Sciences), Heze, China

**Keywords:** antimicrobial packaging, antioxidant packaging, controlled-release systems, food safety, intelligent packaging, migration control, next-generation bio-packaging, shelf-life extension

## Abstract

Next-generation bio-packaging systems employ controlled-release antimicrobial and antioxidant strategies to enhance food preservation while limiting premature depletion of active compounds. Biopolymer matrices and nano-enabled architectures regulate bioactive release through diffusion pathways, encapsulation domains, crystallinity, and matrix organization, thereby influencing preservation efficacy, migration behavior, and food-contact safety. Recent advances demonstrate the effectiveness of engineered antimicrobial systems in controlling microbial proliferation during storage. Hybrid ZnO/graphene oxide/montmorillonite coatings have achieved approximately 60% inhibition of gram-positive and gram-negative microorganisms, highlighting the potential of nano-enabled architectures for sustained antimicrobial protection. Intelligent sensing technologies further support real-time monitoring of freshness and spoilage. Applications across meat, seafood, dairy, fresh produce, bakery, and ready-to-eat foods demonstrate improved shelf life, quality retention, and food safety. Biodegradable materials, renewable feedstocks, and circular-economy compatibility further strengthen the sustainability of these systems. This review examines controlled-release mechanisms, antimicrobial and antioxidant functionalities, intelligent sensing technologies, migration behavior, safety assessment, sustainability considerations, and regulatory perspectives within a unified framework for next-generation bio-packaging systems. Interactions among material structure, bioactive delivery, antimicrobial efficacy, migration characteristics, and food-contact safety determine the performance and application of emerging bio-based packaging technologies. This review integrates controlled-release design, antimicrobial performance, migration control, and food-contact safety to evaluate emerging nano-enabled bio-packaging systems.

## 1 Introduction

Plastic packaging plays a central role in modern food systems because it provides mechanical protection, processing compatibility, and barrier performance during storage and distribution. However, extensive dependence on petroleum-derived plastics has generated significant environmental concerns because these materials persist for long periods after disposal and contribute to microplastic contamination of environmental and biological systems (1, 2). Global plastic production exceeds 400 million tonnes annually, while approximately 130 million tonnes of plastic waste are mismanaged each year, accelerating environmental pollution (3–5). At the same time, food spoilage and quality deterioration continue to generate economic losses exceeding USD 1 trillion annually, whereas foodborne pathogens such as *Staphylococcus aureus*, *Listeria monocytogenes*, *Campylobacter*, and *Salmonella* remain major threats to food safety and public health (6, 7, 94).

These environmental and food-safety challenges have intensified interest in bio-based packaging materials derived from polysaccharides, proteins, lipids, and microbial polyesters. Such materials offer renewable and potentially biodegradable alternatives to conventional plastics, but their broader adoption remains limited by oxygen and moisture permeability, reduced mechanical stability, and insufficient long-term preservation performance (8–10). Polymer-chain mobility and free-volume regions facilitate the transport of gasses, moisture, and active compounds through packaging matrices, thereby reducing their effectiveness during storage. To overcome these limitations, next-generation bio-packaging systems increasingly combine bio-based polymers with controlled-release technologies and nano-enabled functional materials that prolong antimicrobial activity, improve preservation performance, and extend product shelf life (11).

The functionality of these advanced packaging systems is governed by molecular transport processes that regulate the release, distribution, and persistence of active compounds within polymer matrices. Diffusion pathways, encapsulation domains, partitioning behavior, and polymer-bioactive interactions determine the availability of antimicrobial and antioxidant agents throughout storage and distribution (11–13). Naturally derived antimicrobials and inorganic nanomaterials can suppress microbial growth, while nano-enabled delivery platforms improve compound stability and maintain functional activity for extended periods (14–17). Nanostructures additionally modify crystallinity, interfacial organization, and matrix microstructure, thereby influencing molecular transport, barrier performance, and antimicrobial efficacy (10, 18). Figure 1 highlights the major environmental and food-system challenges driving the development of bio-packaging technologies and the role of nanostructures in improving material functionality and sustainability.

**Figure 1:**
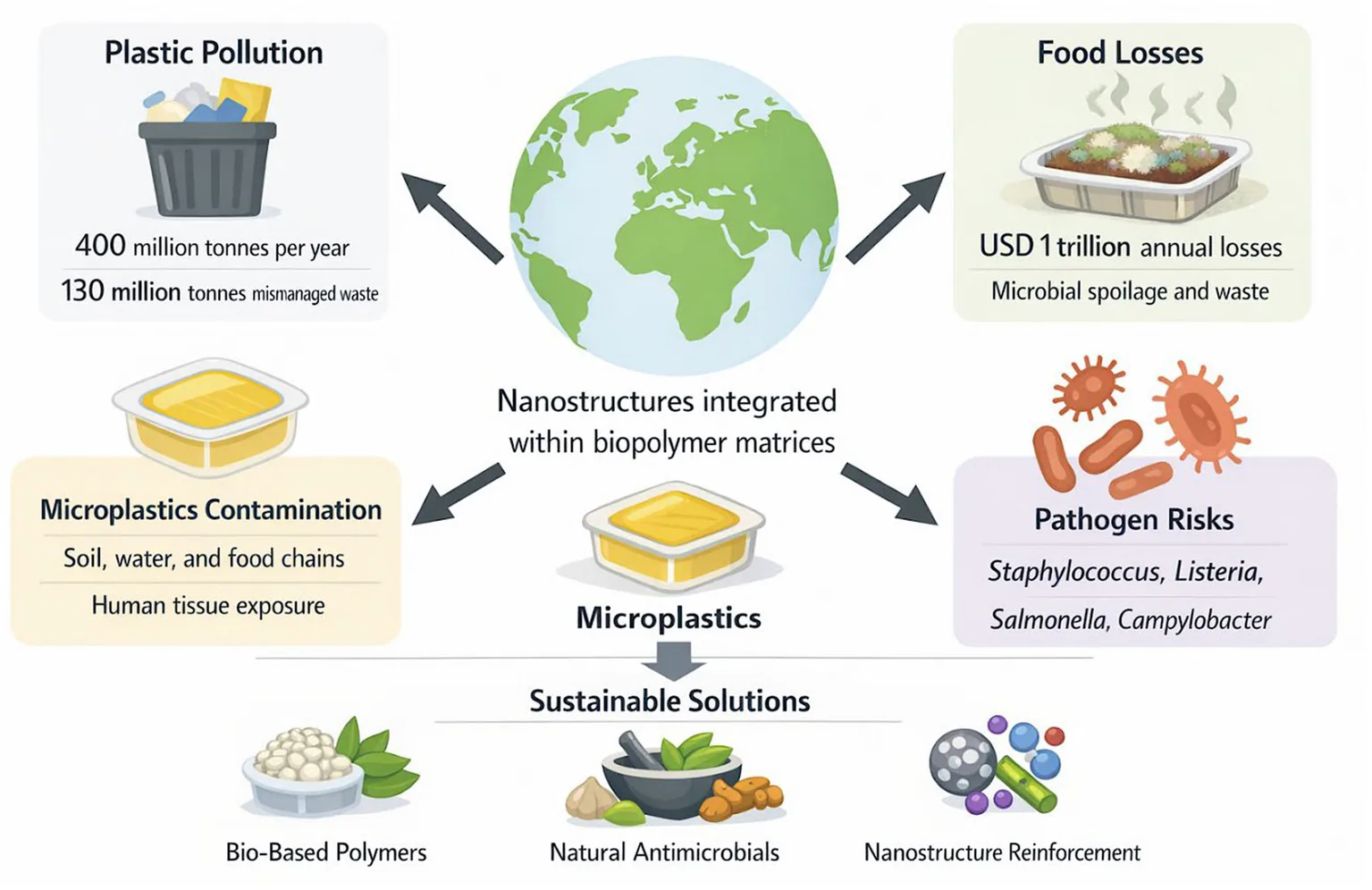
Nanostructures integrated within biopolymer matrices as sustainable solutions to major food-packaging challenges, including plastic pollution, food losses, microbial contamination, pathogen risks, and microplastic generation.

Cellulose nanomaterials, metal-oxide nanoparticles, and hybrid nanofillers increase diffusion-path tortuosity, reduce molecular mobility, and strengthen resistance to oxygen and moisture transfer (19, 95). These structural modifications link material architecture directly with antimicrobial performance and preservation efficacy. At the same time, the transport processes that govern controlled release also influence migration behavior and food-contact safety. Effective packaging design therefore requires a balance between sufficient bioactive availability for microbial control and restricted migration to ensure regulatory compliance and consumer safety (20, 21). Matrix architecture consequently provides the mechanistic connection among bioactive delivery, barrier performance, migration behavior, and food-contact safety in next-generation bio-packaging systems.

This review examines the relationships among material composition, nanostructure design, controlled-release mechanisms, antimicrobial and antioxidant functionality, intelligent sensing technologies, migration behavior, food-contact safety, and sustainability in next-generation bio-packaging systems. Particular emphasis is placed on the role of molecular transport in linking nanostructure engineering with preservation performance, migration control, and practical food-packaging applications. PHBHHx is used as a representative material platform for illustrating the interactions among matrix structure, nanostructure incorporation, and packaging functionality. This review integrates material architecture, active preservation mechanisms, intelligent monitoring technologies, safety assessment, and sustainability considerations within a unified framework for emerging bio-based packaging systems.

### 1.1 Search strategy and inclusion criteria

A structured literature search was conducted using Scopus, Web of Science, PubMed, and Google Scholar. Search terms included antimicrobial packaging, bio-based polymers, nature-derived nanostructures, controlled release, release kinetics, diffusion behavior, migration control, food-contact safety, intelligent indicators, PHBHHx, and ZnO nanostructures. Publications published between 2020 and 2026 were considered. Eligible studies reported quantitative data on antimicrobial activity, release behavior, barrier properties, nanostructure characterization, migration, safety, or green synthesis approaches. Publications lacking experimental validation or clear relevance to food-packaging applications were excluded. Reference lists of key studies were additionally screened to identify relevant publications. Particular emphasis was placed on studies involving PHBHHx-based materials, ZnO nanostructures, controlled-release systems, and sustainable synthesis strategies, including plant-mediated and microbial production routes (2, 22).

### 1.2 Scope of the review and conceptual contribution

This review examines how material architecture regulates antimicrobial performance, controlled release, migration behavior, and food-contact safety in next-generation bio-packaging systems. Coverage includes bio-based polymer matrices, engineered and nature-derived nanostructures, antimicrobial and antioxidant delivery systems, intelligent monitoring technologies, migration-control strategies, sustainability considerations, and regulatory frameworks relevant to food-packaging applications. Particular attention is given to the role of molecular transport in linking nanostructure design with preservation efficacy, barrier performance, and safety outcomes. PHBHHx is used as a representative material platform to illustrate interactions among matrix structure, nanostructure incorporation, and packaging functionality. The principal contribution of this review is the integration of controlled-release mechanisms, antimicrobial functionality, intelligent monitoring, migration assessment, and food-contact safety within a unified framework for next-generation bio-based packaging systems.

## 2 Global drivers for next-generation bio-packaging

Global food systems face increasing demands for food preservation, food safety, resource efficiency, and environmental sustainability. Rising supply-chain complexity, stricter regulatory requirements, and growing consumer expectations have intensified demand for packaging materials that protect food quality while reducing environmental burdens (8, 9). Dependence on petroleum-derived plastics remains a major challenge because of resource depletion, waste accumulation, environmental persistence, and microplastic contamination (1, 4). Consequently, sustainability initiatives and circular-economy strategies are accelerating the transition toward renewable and biodegradable packaging materials with reduced environmental impacts (23, 96). These developments align closely with the United Nations Sustainable Development Goals because improved food preservation contributes to SDG 2 (Zero Hunger), enhanced food safety supports SDG 3 (Good Health and Well-being), and the replacement of conventional plastics supports SDGs 12 and 13 through responsible consumption and reduced environmental pollution (2, 4, 23).

Food spoilage, microbial contamination, and oxidative deterioration continue to generate substantial economic losses throughout food supply chains while compromising product quality and consumer safety (7, 24). Active packaging technologies have therefore emerged as important preservation tools because antimicrobial and antioxidant systems can suppress microbial growth, reduce quality deterioration, and extend shelf life during storage (25, 26). Recent developments have expanded packaging functionality beyond passive containment through the incorporation of renewable biopolymers, bioactive compounds, nano-enabled materials, and controlled-release technologies that maintain preservation efficacy over extended storage periods (27, 28). Controlled-release strategies additionally enable the delivery of antimicrobial compounds into package headspace, extending preservation beyond direct food-contact applications.

Modern packaging systems increasingly integrate protection, preservation, monitoring, and sustainability within a single multifunctional platform. Material design influences barrier performance, product stability, antimicrobial functionality, and storage behavior, while intelligent indicators provide real-time information on freshness, spoilage progression, and product condition throughout distribution networks (27, 29). Environmental pressures, food-safety requirements, sustainability objectives, and technological innovations collectively drive the development of next-generation bio-packaging systems. Bio-based materials, controlled-release antimicrobial technologies, and intelligent monitoring platforms therefore represent complementary approaches for improving food preservation while supporting long-term environmental sustainability. Figure 2 outlines the major drivers promoting the development of next-generation bio-packaging systems and the emerging technologies being adopted to address these challenges.

**Figure 2:**
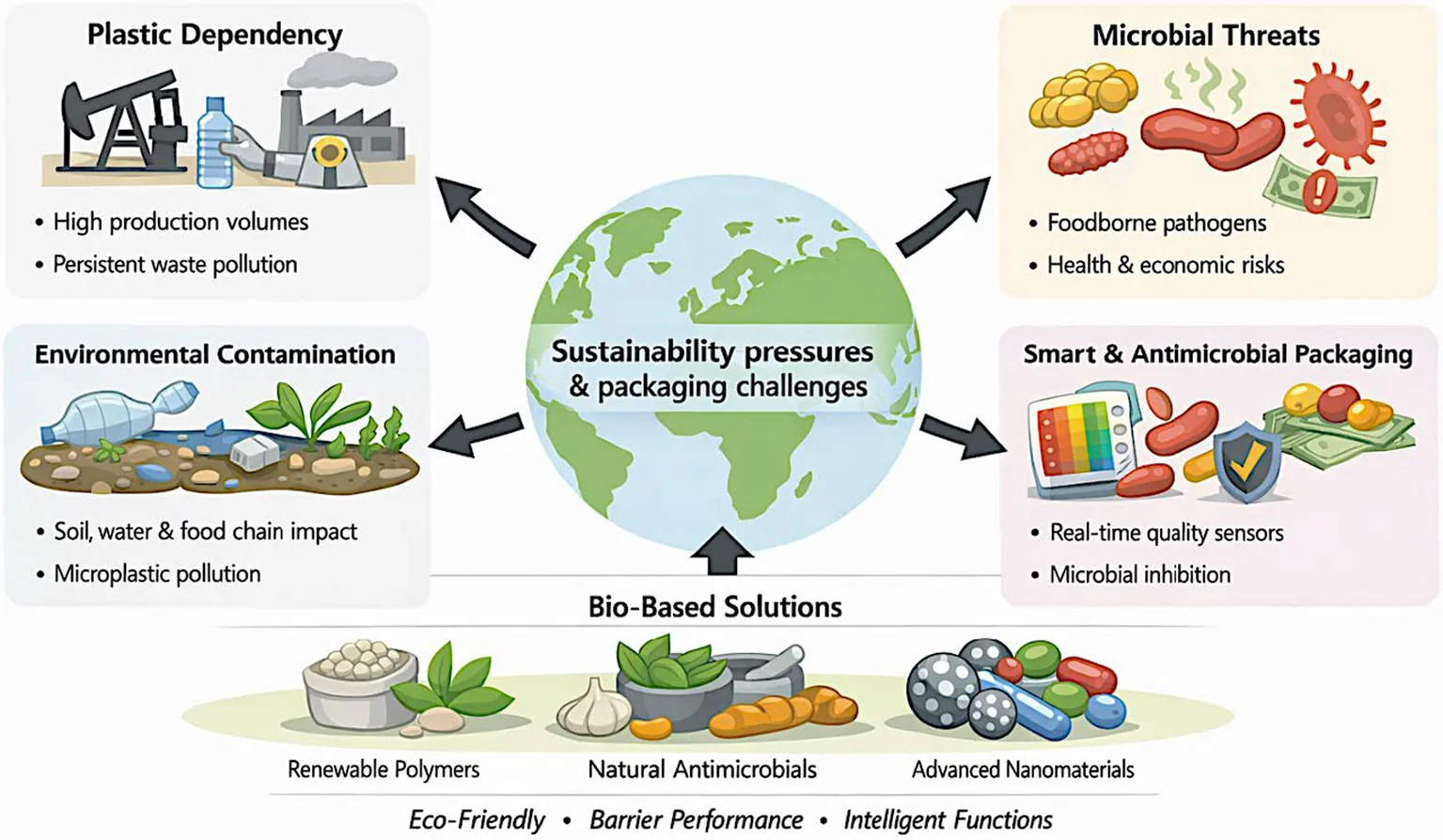
Global drivers, sustainability pressures, and bio-based solutions for next-generation bio-packaging systems.

### 2.1 Major polymer classes and their functional roles

Polysaccharides, proteins, lipids, and microbial polyesters constitute the principal material platforms used in bio-based packaging systems. Their chemical structures determine barrier properties, mechanical performance, moisture sensitivity, molecular transport, and interactions with antimicrobial compounds, thereby influencing packaging functionality under different storage conditions (8–10).

Starch, cellulose, chitosan, alginate, and pectin are among the most widely investigated polysaccharides because dense hydrogen-bonded networks restrict oxygen diffusion and provide favorable oxygen-barrier properties. However, hydrophilic domains increase water absorption and moisture sensitivity under humid conditions (30–33). Chitosan additionally exhibits intrinsic antimicrobial activity through interactions between protonated amino groups and microbial cell membranes, while its compatibility with essential oils and metal-oxide nanoparticles supports improved retention and stability of active compounds (34, 35).

Protein-based materials, including gelatin and zein, provide good compatibility with plant-derived antimicrobial compounds, whereas lipid-based materials such as waxes, fatty acids, and triglycerides improve resistance to water-vapor transmission and strengthen moisture-barrier properties in multilayer systems (36–39). Cellulose nanocrystals and cellulose nanofibrils further enhance structural stability and barrier performance through reinforcement of polymer networks (18, 40).

Polyhydroxyalkanoates (PHAs) have attracted considerable interest because they combine biodegradability, favorable food-contact properties, hydrophobicity, and mechanical robustness (41, 42). Among them, PHBHHx exhibits improved flexibility and processability compared with PHB and PHBV while maintaining desirable barrier characteristics (2, 43). ZnO nanostructures exhibit good compatibility with PHBHHx matrices, where homogeneous nanoparticle dispersion improves barrier properties and antimicrobial functionality. These advantages make PHBHHx-based nanocomposites promising platforms for sustainable antimicrobial packaging applications requiring prolonged functionality and low migration potential (2, 42, 44).

### 2.2 Limitations of conventional bio-based polymers

Despite their sustainability advantages, many bio-based packaging materials exhibit moisture sensitivity, brittleness, limited thermal stability, and variable barrier performance under commercial storage conditions (45, 46). Polysaccharide- and protein-based matrices frequently permit oxygen and water-vapor transmission at levels that accelerate quality deterioration and reduce the long-term effectiveness of active packaging systems. The resulting increase in permeability may also diminish retention of antimicrobial compounds and compromise preservation efficacy during prolonged storage (46).

Natural antimicrobial agents such as essential oils, phenolic compounds, and plant-derived extracts face additional challenges associated with volatility, oxidative degradation, and limited storage stability. Rapid depletion during the early stages of storage frequently reduces antimicrobial availability before the end of product shelf life. Consequently, preservation performance depends not only on the antimicrobial agent itself but also on matrix architecture and delivery-system design (14, 47).

PHAs illustrate the balance required between barrier performance and material processability. PHB exhibits excellent barrier properties because highly ordered crystalline domains restrict molecular transport, although high crystallinity also increases brittleness and limits processing flexibility (41, 42). PHBHHx addresses several of these limitations through lower crystallinity, which improves flexibility while maintaining favorable barrier behavior (2). The incorporation of ZnO nanostructures further modifies matrix organization, reduces molecular mobility, and restricts diffusion pathways, thereby contributing to enhanced barrier performance, improved storage stability, and lower migration potential (10, 44, 48).

### 2.3 Material innovations and functional improvement

Nanostructures have emerged as effective tools for overcoming many limitations associated with biodegradable packaging materials. The incorporation of nanoparticles and bioactive fillers modifies matrix architecture, molecular mobility, and interfacial organization, resulting in improved mechanical performance, enhanced moisture resistance, and stronger antimicrobial functionality (49, 50).

Nano-reinforced matrices frequently exhibit greater tensile strength, reduced water-vapor permeability, and improved retention of active compounds during storage. Interactions between nanofillers and polymer chains restrict chain mobility, improve stress distribution, and increase resistance to structural deterioration. Well-dispersed fillers increase diffusion-path complexity within the matrix, reducing oxygen and moisture transport while delaying quality deterioration (19).

Encapsulation systems and nano-enabled delivery platforms provide additional functional benefits by protecting essential oils, phenolic compounds, and other natural antimicrobials from volatilization and oxidative degradation. Physical confinement within nano-scale carriers improves compound stability and preserves antimicrobial functionality during storage. Controlled release of bioactive compounds prolongs antimicrobial and antioxidant activity, maintains availability at food-contact interfaces, and improves preservation efficacy compared with direct incorporation of free active agents (49).

Processing technologies including electrospinning, extrusion, solvent casting, coating, and multilayer fabrication further influence nanostructure dispersion, matrix organization, and packaging performance. These approaches support the development of multifunctional packaging systems that combine barrier properties, antimicrobial activity, and intelligent monitoring capabilities within a single material platform (27, 51–53).

### 2.4 Sustainability drivers and circular bio-based feedstocks

Agricultural residues, microbial biopolymers, and waste-valorization streams provide renewable feedstocks for sustainable packaging materials (54–56). Polysaccharides, natural fibers, and functional additives recovered from agricultural by-products serve as valuable raw materials for biodegradable composites, while microbial fermentation processes generate high-performance biopolymers at industrial scale and expand the availability of renewable packaging materials (54, 55). PHAs represent an important class of microbial biopolymers because their biodegradability, tunable mechanical properties, and adaptable barrier behavior support applications in active-packaging systems (41, 42). Renewable feedstocks used for PHA production further strengthen their suitability within circular-bioeconomy frameworks.

Fermentation technologies, polymer-blending approaches, and surface-coating strategies influence both material performance and production efficiency (42, 57). Fermentation processes increase polymer yields, whereas blending and coating strategies enhance barrier performance and expand material functionality. Reported studies indicate increases in PHA yields of approximately 60% together with barrier-performance improvements approaching 35% (42).

Sustainable feedstocks, circular material flows, and active-preservation functions collectively contribute to the development of modern packaging systems that reduce dependence on fossil-derived resources and lower environmental burdens across the packaging life cycle (23, 53, 55). The growing integration of renewable polymers with antimicrobial and barrier-enhancing nanostructures highlights the potential of circular bio-based materials to simultaneously address preservation, sustainability, and food-safety requirements (6, 56).

### 2.5 Antimicrobial preservation and intelligent monitoring

Food quality depends on effective microbial control and reliable assessment of product status throughout storage and distribution. Antimicrobial packaging systems inhibit microbial growth at food surfaces and delay spoilage processes that reduce product quality and shelf life (27, 29). Intelligent packaging technologies complement these preservation functions by monitoring freshness, spoilage progression, and storage conditions. pH-responsive pigments, gas-sensitive indicators, and temperature-responsive colorimetric systems generate visible responses to physicochemical changes occurring within packaged foods (28, 29). Beetroot-derived films, for example, detect freshness changes in refrigerated fish through pH-dependent color transitions, demonstrating the growing role of intelligent monitoring systems in modern food packaging (58).

Recent developments in bio-based plastics and polymer nanocomposites have expanded packaging functionality beyond conventional barrier protection. Antimicrobial agents, oxygen scavengers, freshness indicators, biosensors, and colorimetric monitoring platforms can be integrated within multifunctional packaging materials, while nanocomposite reinforcement improves barrier, mechanical, thermal, and antimicrobial properties. Hybrid ZnO/graphene oxide/montmorillonite coatings exemplify this multifunctional approach by integrating barrier enhancement with active antimicrobial functionality within a single packaging architecture, demonstrating the versatility of nanocomposite-based food-packaging systems (59).

High-barrier multilayer packaging films with oxygen transmission rates as low as 3.95 cm^3^/m^2^/day reduced microbial growth, decreased the relative abundance of *Pseudomonas*, and extended the shelf life of refrigerated yak meat to 20 days, demonstrating the close relationship between barrier performance and microbial control in modern food-packaging systems (97). These findings illustrate how antimicrobial functionality, barrier enhancement, and material design collectively improve food preservation and reduce spoilage during storage.

## 3 Material platforms in bio-packaging systems

Bio-based material classes provide the structural foundation for next-generation packaging systems. Polymer composition and matrix architecture influence material properties relevant to food-packaging applications, including mechanical stability, barrier behavior, compatibility with active compounds, and food-contact performance (9, 10). Biodegradable alternatives to petroleum-derived plastics have attracted increasing attention because renewable feedstocks and biodegradable end-of-life pathways align with sustainability objectives (23).

Polysaccharides, proteins, lipids, and microbial polyesters differ substantially in structural characteristics and functional properties. Material selection influences packaging performance, processing requirements, active-compound incorporation, and application suitability under different storage conditions (8, 10). Figure 3 displays the major material platforms used in next-generation bio-packaging systems and their principal functional characteristics.

**Figure 3:**
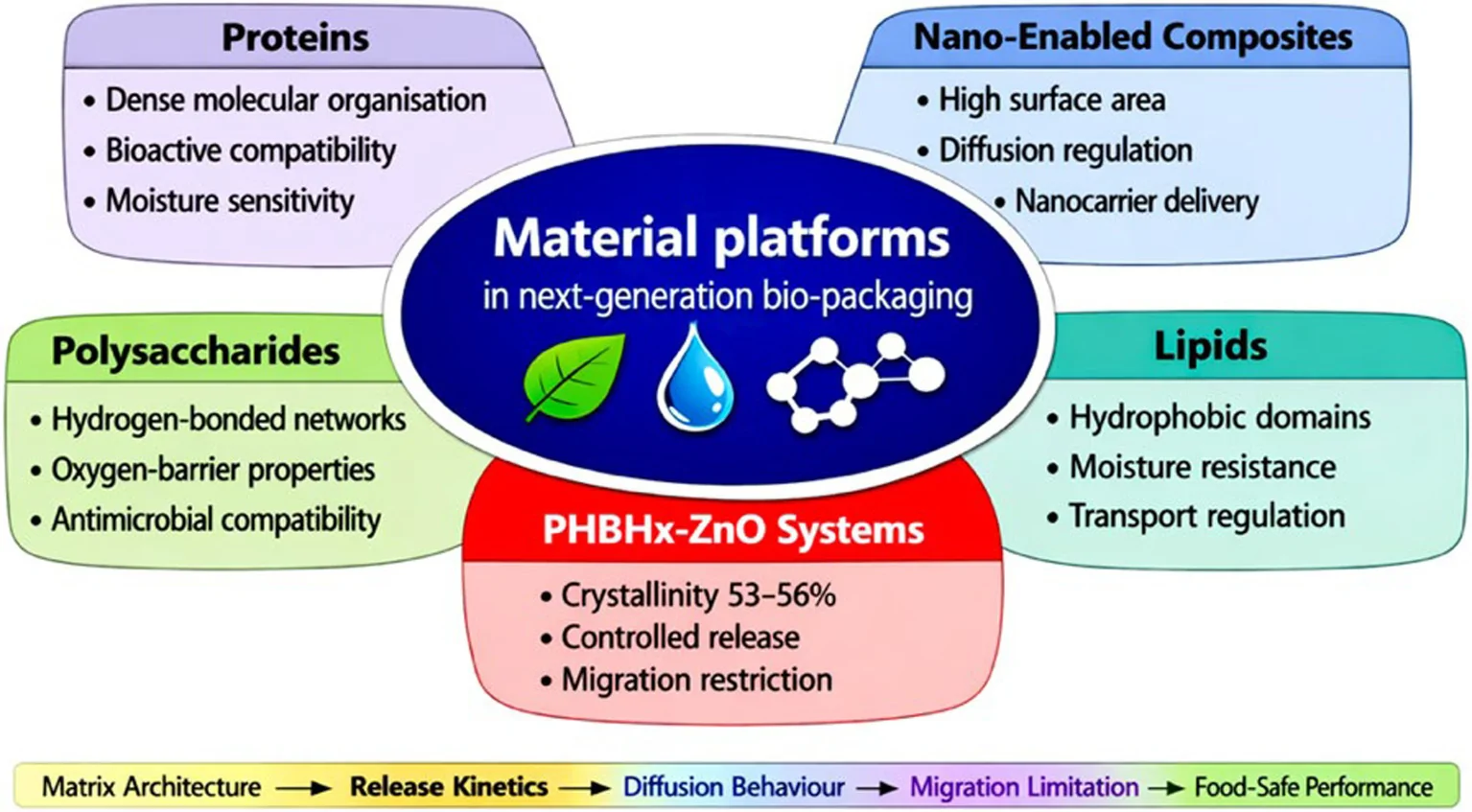
Major material platforms and functional characteristics of next-generation bio-packaging systems.

### 3.1 Biopolymer structural matrices

Biopolymer-based materials provide the structural foundation for next-generation food-packaging systems. Renewable feedstocks, biodegradability, and compatibility with active compounds distinguish these materials from conventional petroleum-derived plastics (9, 60). Polymer composition influences barrier behavior, mechanical stability, environmental durability, and food-contact performance (10, 23). Polysaccharides represent the most widely used biopolymer class in food-packaging applications. Starch, cellulose, chitosan, alginate, and pectin form biodegradable films with favorable oxygen-barrier properties arising from dense hydrogen-bonded networks (30, 33). Moisture sensitivity arises from water absorption, which increases molecular mobility and permeability under humid conditions (31, 45).

Chitosan occupies a distinct position among polysaccharide-based materials because protonated amino groups interact with microbial cell membranes and provide intrinsic antimicrobial activity (34, 61). Film-forming ability and compatibility with bioactive compounds further support application in active-packaging systems (35). Protein-based matrices, including gelatin, zein, soy protein, and whey protein, exhibit favorable oxygen-barrier characteristics arising from compact molecular organization and extensive intermolecular interactions (38, 39). Moisture uptake can reduce barrier stability under high-humidity storage conditions (10). Lipid-based materials, including waxes, fatty acids, and triglycerides, provide effective resistance to water-vapor transfer (36, 37). Composite systems combining proteins and lipids achieve balanced moisture resistance and gas-barrier performance through complementary material characteristics (36).

### 3.2 Microbial polyesters and PHBHHx-ZnO materials

Microbial polyesters, particularly PHAs, combine biodegradability with favorable mechanical and barrier properties suitable for food-packaging applications (41, 42). Semi-crystalline polymer structures restrict gas and moisture diffusion and contribute to effective oxygen-barrier performance in food-contact materials (62). Compatibility with renewable feedstocks and biodegradable end-of-life pathways further increases interest in PHAs as sustainable packaging materials (42). Poly(3-hydroxybutyrate) (PHB) exhibits excellent oxygen-barrier properties because highly ordered crystalline domains restrict gas diffusion (41). High crystallinity, however, increases brittleness and limits processing flexibility in some packaging applications (10).

PHBHHx addresses this limitation through the incorporation of hydroxyhexanoate units that reduce crystallinity and increase toughness and flexibility (43). Greater hydrophobicity also reduces water uptake and increases dimensional stability under food-storage conditions (42, 43). ZnO nanostructures provide an effective reinforcement platform for PHBHHx matrices. Interactions between ZnO surfaces and PHBHHx chains modify crystallinity and reduce molecular mobility within the polymer network (43, 44).

Nanoparticle incorporation improves barrier properties, ultraviolet shielding, and mechanical stability while maintaining the biodegradability of the underlying polyester system (43, 48). These characteristics support the development of multifunctional bio-packaging systems based on PHBHHx-ZnO nanocomposites. ZnO incorporation not only improves barrier and mechanical performance but also introduces antimicrobial functionality, making PHBHHx-ZnO systems representative examples of multifunctional bio-nanocomposite platforms that combine structural reinforcement, preservation efficacy, and sustainable material design (2, 44).

### 3.3 Nano-enabled bio-nanocomposite materials

Nano-enabled bio-nanocomposite materials modify polymer structure through the incorporation of nanoscale fillers. Cellulose nanocrystals, cellulose nanofibrils, metal-oxide nanoparticles, and layered silicates influence crystallinity, interfacial organization, free-volume distribution, and matrix stability through interactions with polymer chains (18, 40). Structural changes arising from these interactions influence mechanical performance, barrier behavior, and material durability. Nanocellulose-based fillers form dense hydrogen-bonded networks within polymer matrices. Network formation restricts diffusion through the polymer network and increases tensile strength. Compact matrix organization limits oxygen and moisture permeation, strengthening barrier performance during storage (19, 40). Homogeneous filler distribution increases matrix uniformity and contributes to consistent material performance (49).

Metal-oxide nanoparticles provide both reinforcement and functional activity within bio-nanocomposite systems. ZnO, TiO_2_, and silver nanoparticles influence polymer organization through nanoparticle-matrix interactions that modify crystallinity and diffusion behavior (16, 17). The incorporation of graphene oxide and montmorillonite alongside ZnO nanoparticles has also been shown to improve coating performance through synergistic interactions between inorganic fillers, producing nanostructured materials with enhanced packaging functionality (59). These examples illustrate the multifunctional potential of engineered nanocomposite coatings for food-packaging systems.

Layered silicates and nanoclays alter matrix architecture through intercalated or exfoliated arrangements. Greater diffusion-path complexity restricts the transport of gasses and water vapor through the polymer network. Lower permeability arises from these structural characteristics (10). Nanocarriers, liposomes, core-shell structures, and microcapsules provide protective environments for incorporated bioactive compounds. Physical separation from the surrounding environment limits volatilization and degradation during storage (14, 15). Material performance within these systems depends on particle size, surface properties, matrix compatibility, and distribution of nanostructures within the polymer network (10, 18).

### 3.4 Matrix design for antimicrobial delivery and migration control

Matrix architecture regulates antimicrobial availability and migration behavior within active packaging systems (10, 20). Polymer crystallinity, porosity, compatibility, and environmental conditions influence diffusion behavior and mass transfer through packaging materials (20, 21). Diffusion and partitioning processes determine the movement of active compounds within polymer matrices and between packaging materials and food systems (20).

Polymer domains, microcapsules, and nanocarriers localize antimicrobial compounds within protected regions of the matrix. Physical confinement reduces premature degradation and slows antimicrobial depletion during storage (14, 15). Core-shell structures and nanofibrous architectures further regulate diffusion and reduce rapid initial release of active compounds (13, 52). Multilayer packaging systems distribute barrier and antimicrobial functions across separate structural layers (63). Barrier layers restrict diffusion and migration, whereas active layers provide localized antimicrobial activity. Spatial separation of these functions enables antimicrobial delivery while limiting excessive transfer of active compounds into food systems (63, 64).

Polymer chemistry strongly influences transport behavior. Hydrophobic polymers, including PHBHHx, reduce water uptake and limit molecular mobility within the matrix (2, 10). Hydrophilic matrices exhibit greater moisture absorption and higher diffusion under humid conditions (45, 46). Lipid incorporation and polymer blending modify transport characteristics according to packaging requirements (36, 37). Stimuli-responsive materials provide additional control over antimicrobial delivery. Changes in pH, temperature, humidity, and enzymatic activity alter matrix permeability and influence release behavior (12, 13). Matrix design determines antimicrobial availability and migration behavior within active-packaging systems through its effects on molecular transport, crystallinity, interfacial compatibility, and nanostructural organization during storage and food contact (20, 21).

## 4 Nature-derived nanostructures and green synthesis strategies

Nature-derived nanostructures provide renewable alternatives to conventional nanomaterials while contributing mechanical reinforcement, barrier enhancement, bioactive delivery, and antimicrobial functionality within bio-based packaging systems. Nanoscale dimensions influence morphology, surface chemistry, dispersion behavior, and interfacial interactions, thereby affecting mechanical performance, molecular transport, and material stability within biodegradable matrices (65, 66). Plant-derived, microbial, and polysaccharide-based nanostructures represent important components of next-generation packaging materials because their interactions with polymer networks modify matrix organization, restrict permeability, and improve resistance to environmental stress (16, 18).

Green synthesis approaches utilize biological resources and environmentally compatible processing routes for nanomaterial production. Plant extracts, microbial metabolites, and naturally derived polymers provide renewable alternatives to conventional synthesis methods while reducing environmental burden and supporting resource efficiency (22, 65). Figure 4 illustrates the principal classes of nature-derived nanostructures, representative green synthesis routes, and major functions within next-generation bio-packaging materials.

**Figure 4:**
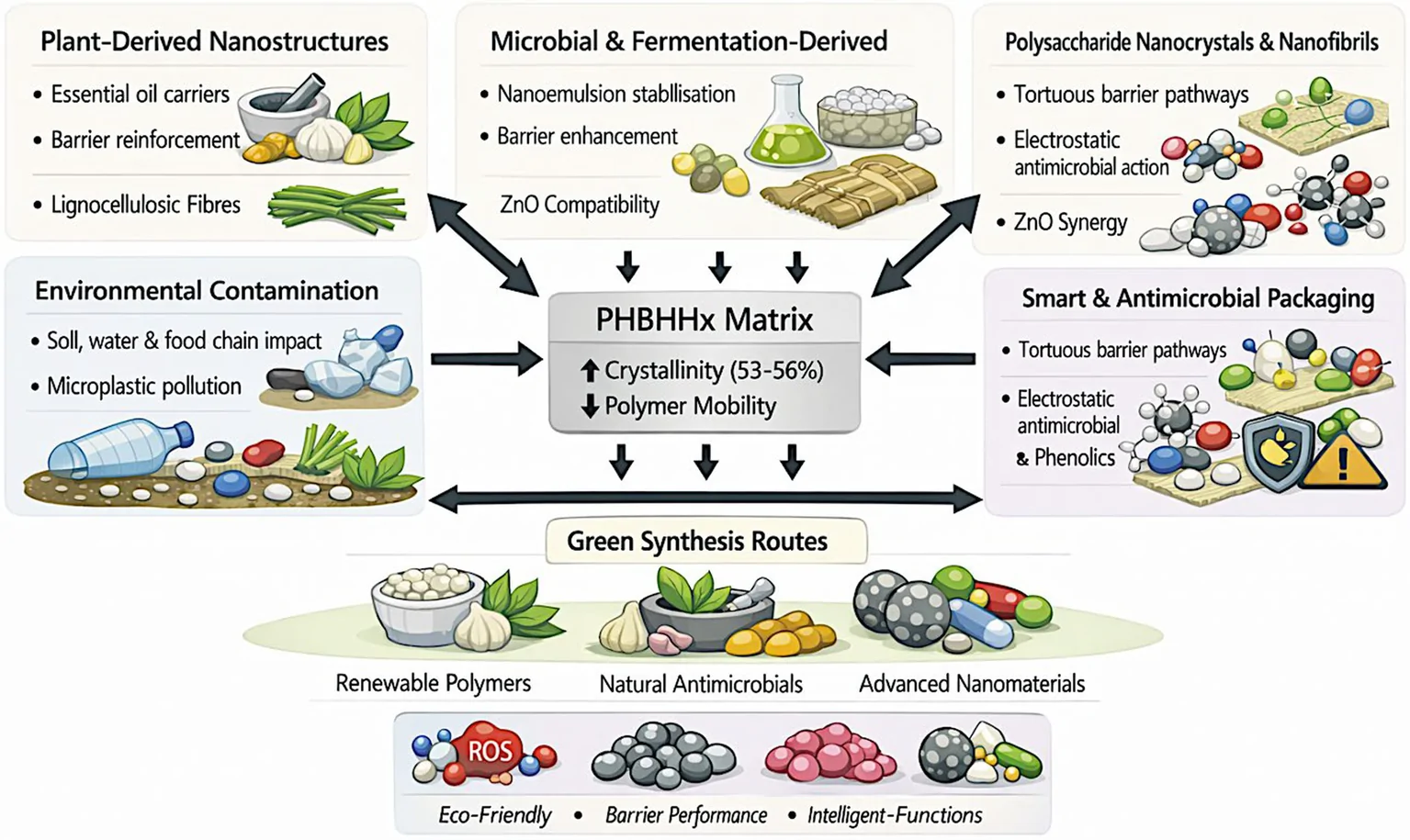
Nature-derived nanostructures, green synthesis routes, and functional roles in next-generation bio-packaging systems.

### 4.1 Plant-derived nanostructures

Plant-derived nanostructures utilize renewable biomass as a source of functional nanomaterials and green-synthesis precursors for bio-packaging applications. Plant extracts provide reducing and stabilizing compounds during the synthesis of metal and metal-oxide nanoparticles, reducing dependence on conventional chemical reagents and influencing particle characteristics (25, 54). Essential oils incorporated within nanoscale carrier systems exhibit greater storage stability than free compounds. Protective carrier structures reduce volatilization and limit degradation of bioactive constituents during storage (12, 14).

Agricultural residues provide a valuable source of lignocellulosic nanofibers for packaging applications. High-aspect-ratio fibers reinforce polymer matrices through strong interfacial interactions and increased network density. Network formation increases mechanical strength and restricts the transport of gasses and moisture through the matrix (19, 55). Matrix organization influences the effectiveness of lignocellulosic reinforcement. Uniform nanofibre dispersion increases interfacial contact and promotes homogeneous material structures (18, 49). Renewable feedstocks recovered from agricultural residues also support resource valorisation within circular bioeconomy systems (56, 57).

### 4.2 Microbial and fermentation-derived nanostructures

Microbial and fermentation-derived nanostructures originate from controlled biological production processes and exhibit good compatibility with bio-based packaging materials. Microbial biosurfactants stabilize nanoemulsions containing hydrophobic bioactive compounds and improve their distribution within hydrophilic matrices (67, 68). Stable nanoscale dispersions reduce droplet coalescence and increase formulation uniformity during storage (12).

Bacterial cellulose forms highly crystalline nanofibrillar networks with strong reinforcing capacity. Dense nanofibrillar structures strengthen matrix integrity and barrier performance in biodegradable packaging materials (69, 70). Nanofibril organization restricts diffusion through the polymer network and contributes to structural stability (18). Microbial fermentation processes provide renewable production routes for functional materials used in advanced packaging systems. Agricultural residues, industrial by-products, and other renewable carbon sources serve as raw materials for these processes and reduce dependence on fossil-derived resources (54, 56).

### 4.3 Polysaccharide nanocrystals and nanofibrils

Cellulose nanocrystals, cellulose nanofibrils, chitin nanocrystals, and chitosan nanoparticles represent the principal polysaccharide-derived nanostructures used in bio-packaging systems. Highly organized nanoscale networks strengthen polymer matrices and restrict oxygen and water-vapor transmission through extended diffusion pathways (40, 71). Dense nanoscale networks strengthen barrier performance and mechanical stability through formation of extended diffusion pathways. Chitosan nanoparticles provide a distinct functional advantage among polysaccharide nanostructures. Cationic surface groups interact with microbial cell membranes and disrupt membrane integrity, leading to growth inhibition (34, 72). Compatibility with a wide range of active packaging materials further supports the application of chitosan nanoparticles in antimicrobial systems. Polysaccharide nanocrystals strengthen polymer-particle interactions and influence nanostructure distribution within composite matrices (18). Interfacial organization contributes to matrix uniformity and material stability (65).

### 4.4 Green synthesis routes and circular feedstocks

Plant extracts, microbial systems, enzymatic processes, and agricultural residues provide the basis for green nanomaterial production in bio-packaging applications (47, 54). Naturally derived reducing agents are replacing many conventional chemical reagents, thereby reducing the environmental burden associated with nanomaterial synthesis (23, 66). Plant-mediated synthesis offers an environmentally friendly route for producing metal and metal-oxide nanoparticles. Plant-derived reducing agents can influence nanoparticle size, morphology, surface chemistry, and antimicrobial performance, enabling the production of functional nanomaterials suitable for active food-packaging applications (22, 73). Synthesis conditions subsequently influence particle size, morphology, and surface characteristics, which affect nanomaterial behavior within packaging matrices (17, 73).

Agricultural waste streams provide abundant feedstocks for lignocellulosic nanomaterials and other bio-derived packaging components (31, 49). Conversion of crop residues and agro-industrial by-products into functional materials supports resource valorisation while reducing disposal burdens and dependence on virgin raw materials (54, 56). Circular production systems link renewable feedstocks, resource valorisation, and functional material manufacturing within a unified sustainability framework. These attributes position green-synthesis strategies as important contributors to the development of environmentally responsible bio-packaging materials (28, 65).

Nature-derived nanostructures and green-synthesis strategies provide multiple functional advantages in bio-based packaging systems, including reinforcement of matrix architecture, enhancement of barrier properties, incorporation of antimicrobial activity, controlled bioactive delivery, and ultraviolet shielding (16, 18, 22, 40). Plant-derived nanomaterials, microbial nanostructures, polysaccharide-based nanoparticles, green-synthesized metal-oxide nanomaterials, and bioactive nanocarriers contribute complementary functionalities that improve packaging performance through modifications of matrix architecture, diffusion behavior, and interfacial interactions (18, 22, 34, 40, 70). Table 1 summarizes representative nanostructures, their sources or synthesis routes, principal functional properties, and packaging applications.

**Table 1:** Representative nature-derived nanostructures, green synthesis routes, and packaging applications.

Nanostructure	Source/Synthesis route	Principal functional properties	Representative packaging applications	References
Cellulose nanocrystals	Plant biomass	Mechanical reinforcement, oxygen-barrier enhancement, diffusion-path tortuosity	Bio-nanocomposite films and coatings	(18, 40)
Cellulose nanofibrils	Plant biomass	Tensile strengthening, moisture-barrier improvement	Reinforced biodegradable packaging materials	(19, 49)
Bacterial cellulose	Microbial fermentation	Highly crystalline network, mechanical stability, barrier enhancement	Active and intelligent packaging systems	(69, 70)
Chitosan nanoparticles	Chitin/chitosan-derived systems	Antimicrobial activity, bioactive delivery, matrix reinforcement	Antimicrobial food packaging	(34, 72)
Lignocellulosic nanofibers	Agricultural residues	Mechanical reinforcement, permeability reduction	Sustainable composite packaging films	(55, 56)
Green-synthesized ZnO nanostructures	Plant-mediated synthesis	Antimicrobial activity, UV shielding, barrier enhancement	Active packaging materials	(16, 22)
Nanoemulsion-based bioactive carriers	Microbial and plant-derived systems	Controlled release, protection of volatile compounds	Active antimicrobial packaging	(12, 14)

## 5 Controlled-release antimicrobial systems

Controlled-release systems regulate the release of antimicrobial compounds from packaging materials throughout food storage. Premature depletion of antimicrobial agents shortens protection periods in conventional active-packaging systems and reduces preservation efficacy before the end of product shelf life (11, 12). Essential oils, phenolic compounds, and other natural antimicrobials often exhibit volatility and limited storage stability. Encapsulation systems and protective carriers reduce volatilization and degradation of active compounds during storage (14, 47). Compound affinity, solubility, matrix structure, and storage conditions influence antimicrobial availability at food surfaces (11, 12).

Diffusion-controlled systems represent the most widely used controlled-release approach in antimicrobial packaging. Diffusive movement through polymer matrices governs the availability of active compounds during storage and determines the duration of antimicrobial protection. Matrix hydration governs antimicrobial availability in swelling-controlled systems, whereas matrix degradation generates antimicrobial release during progressive structural breakdown (13). Diffusion rate, matrix structure, and carrier composition collectively determine antimicrobial availability and preservation performance during storage (11, 12). Antimicrobial release must provide sustained preservation activity without promoting excessive migration of active substances into food products. Figure 5 presents the principal mechanisms responsible for controlled antimicrobial release within bio-packaging materials through diffusion-controlled, swelling-controlled, and degradation-mediated transport pathways.

**Figure 5:**
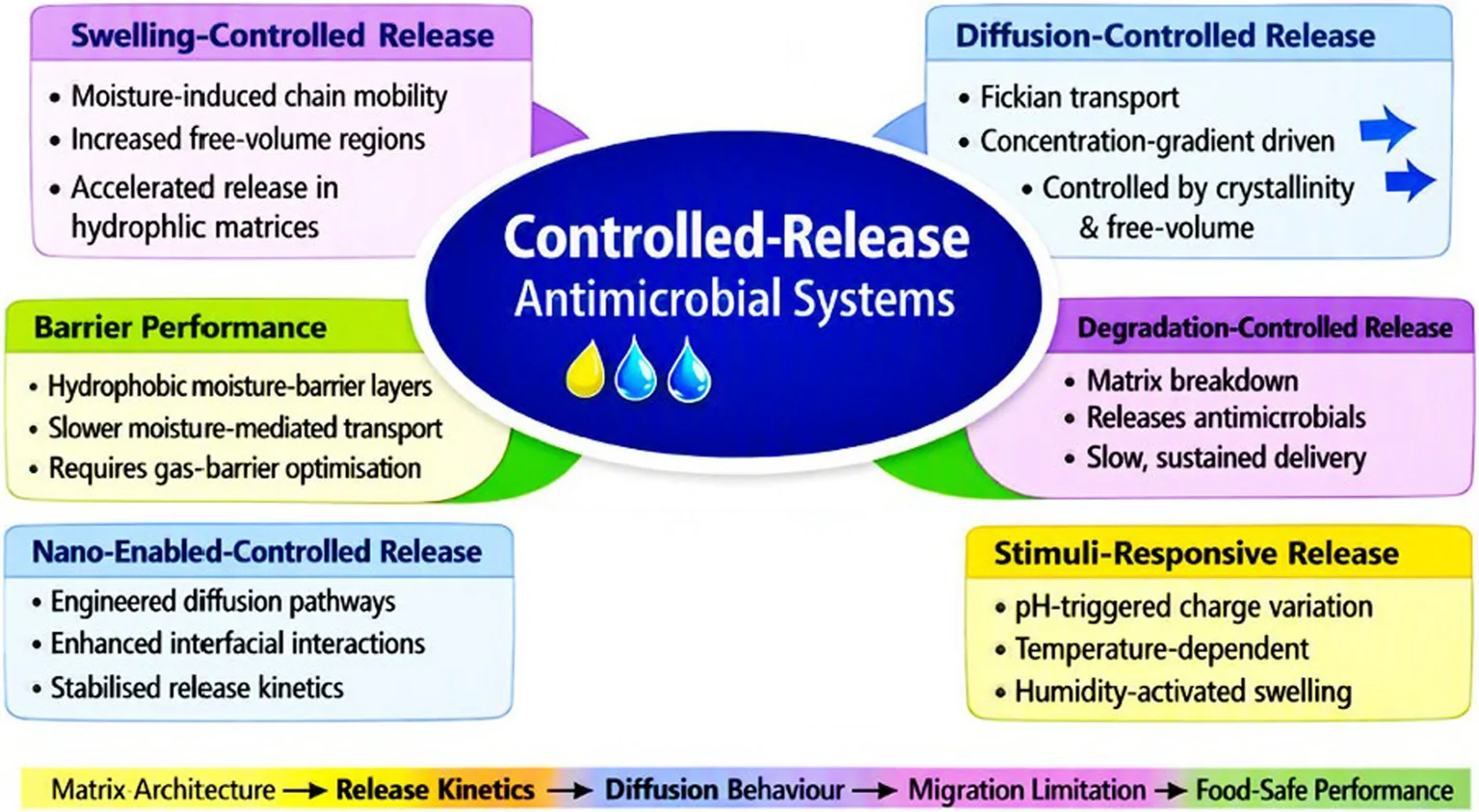
Mechanisms controlling the release of antimicrobial compounds in bio-packaging systems.

### 5.1 Fundamental mechanisms of controlled release

Concentration gradients drive antimicrobial release from active-packaging materials and determine the movement of bioactive compounds from polymer matrices toward food-contact interfaces (11, 13). Diffusion represents the dominant release mechanism in many packaging systems and governs the rate at which active compounds become available during storage (11, 13). Release behavior depends on polymer structure, compound solubility, and interactions between the matrix and the active agent (12). Water uptake provides an additional release mechanism in hydrophilic materials because matrix swelling increases internal free volume and facilitates movement of entrapped compounds through the polymer network (45, 46).

Polysaccharide-based materials often display pronounced humidity-dependent release behavior because moisture absorption alters matrix structure and transport characteristics (45). Polymer degradation provides a third release pathway, whereby progressive matrix breakdown exposes previously entrapped compounds and increases their release over time (10, 64). Diffusion, swelling, and degradation frequently operate simultaneously within active-packaging materials, and their combined effects determine antimicrobial release behavior throughout storage (11, 12).

Vapor-phase delivery systems extend the principles of diffusion-controlled release by transporting antimicrobial agents into package headspace rather than directly onto food surfaces. Antimicrobial efficacy therefore depends not only on release from the packaging matrix but also on evaporation, adsorption, gas-phase diffusion, and distribution within the package environment. Mathematical modeling of carvacrol transport demonstrated that these processes, together with package geometry, govern headspace concentration profiles and antimicrobial availability over time (74). Such approaches provide a quantitative framework for designing non-contact antimicrobial packaging systems and optimizing preservation efficacy during storage.

### 5.2 Nano-enabled controlled-release systems

Nano-enabled delivery systems protect antimicrobial compounds and increase retention within active-packaging materials. Liposomes, nanoemulsions, polymeric nanocarriers, and core-shell structures separate bioactive compounds from the surrounding environment and reduce losses arising from volatilization and degradation (14, 15). Carrier structure determines both the timing and extent of antimicrobial release. Physical confinement within nanoscale domains limits rapid depletion of active compounds, whereas protection from volatilization and degradation preserves antimicrobial functionality throughout storage (12, 13).

Nanostructured fillers modify matrix organization and permeability, thereby influencing release kinetics and bioactive availability within packaging materials (18). Release behavior consequently depends on interactions between carrier characteristics and matrix structure. Hydrophobic antimicrobial compounds often distribute more uniformly within hydrophilic matrices when nanoscale carrier systems provide physical separation from the surrounding polymer network. Improved distribution promotes more uniform release profiles, reduces localized depletion of active compounds, and supports sustained antimicrobial functionality throughout storage (12, 13).

### 5.3 Stimuli-responsive controlled-release systems

Stimuli-responsive packaging materials regulate antimicrobial release according to changes within the food environment. Variations in pH, temperature, humidity, light exposure, and enzymatic activity alter matrix permeability and molecular transport through swelling, bond cleavage, and charge modification (12, 13). pH-responsive materials release antimicrobial compounds when changes in acidity modify polymer-charge interactions (13).

Temperature fluctuations alter diffusion rates within polymer matrices and influence the transport of active compounds under changing storage conditions (12). Humidity-responsive systems accelerate diffusion through water absorption. Water uptake increases polymer-chain mobility, expands free-volume regions, and accelerates movement of encapsulated compounds under conditions favorable for microbial growth (13, 64).

Responsive packaging platforms frequently combine multiple environmental triggers within a single material system. Simultaneous responses to pH, moisture, temperature, and enzymatic activity generate release profiles matched to storage conditions and product status (12, 13).

### 5.4 Intelligent monitoring and responsive antimicrobial delivery

Physicochemical changes occurring within packaged foods influence antimicrobial release throughout storage. Headspace composition, gas exchange, microbial activity, and changes in product quality modify conditions surrounding active packaging materials and affect antimicrobial delivery systems (27, 53).

Intelligent packaging technologies monitor pH, temperature, gas composition, and other spoilage-related indicators, providing real-time information on product status during storage. Indicators detect changes associated with spoilage progression, whereas responsive packaging materials adjust antimicrobial delivery according to prevailing environmental conditions. The combination of monitoring and controlled-release functions enables simultaneous assessment of food quality and preservation status within a single packaging platform. Integration of these functions supports maintenance of sensory quality, suppression of microbial growth, and improved shelf-life management throughout storage and distribution (27, 28).

## 6 Antimicrobial mechanisms and functional activity

Antimicrobial compounds suppress microbial growth through interactions with microbial membranes, intracellular components, and metabolic processes. Membrane disruption, oxidative damage, ion release, and enzyme inhibition represent the principal mechanisms responsible for microbial inactivation in nano-enabled bio-packaging materials (13, 75). Structural and functional damage within microbial cells reduces spoilage and supports food preservation during storage. Antimicrobial activity depends on both active-compound properties and delivery-system characteristics. Compound stability, availability at the food surface, and contact with microbial cells influence the extent and duration of microbial control (12, 14). Nanoscale carriers increase compound dispersion and contact with microbial targets, influencing antimicrobial performance (12, 15).

ZnO nanostructures represent one of the most widely studied antimicrobial nanomaterials for food-packaging applications. Reactive oxygen species (ROS) generated at nanoparticle surfaces damage microbial membranes, proteins, and nucleic acids (16). Zn^2+^ ions further disrupt membrane function and cellular metabolism, leading to inhibition of microbial growth (98). Antimicrobial activity arises from the combined action of oxidative and ionic mechanisms. Microbial susceptibility varies according to cell structure, physiological state, and environmental conditions. Antimicrobial effectiveness consequently differs among microbial species and food systems (76). Sustained antimicrobial performance during storage depends on continued availability of active compounds at microbial interfaces and their interaction with susceptible cellular targets. Figure 6 maps the principal pathways responsible for antimicrobial functionality in nano-enabled bio-packaging materials, including membrane disruption, oxidative stress, ion release, diffusion-controlled delivery, encapsulation, and polymer-mediated transport.

**Figure 6:**
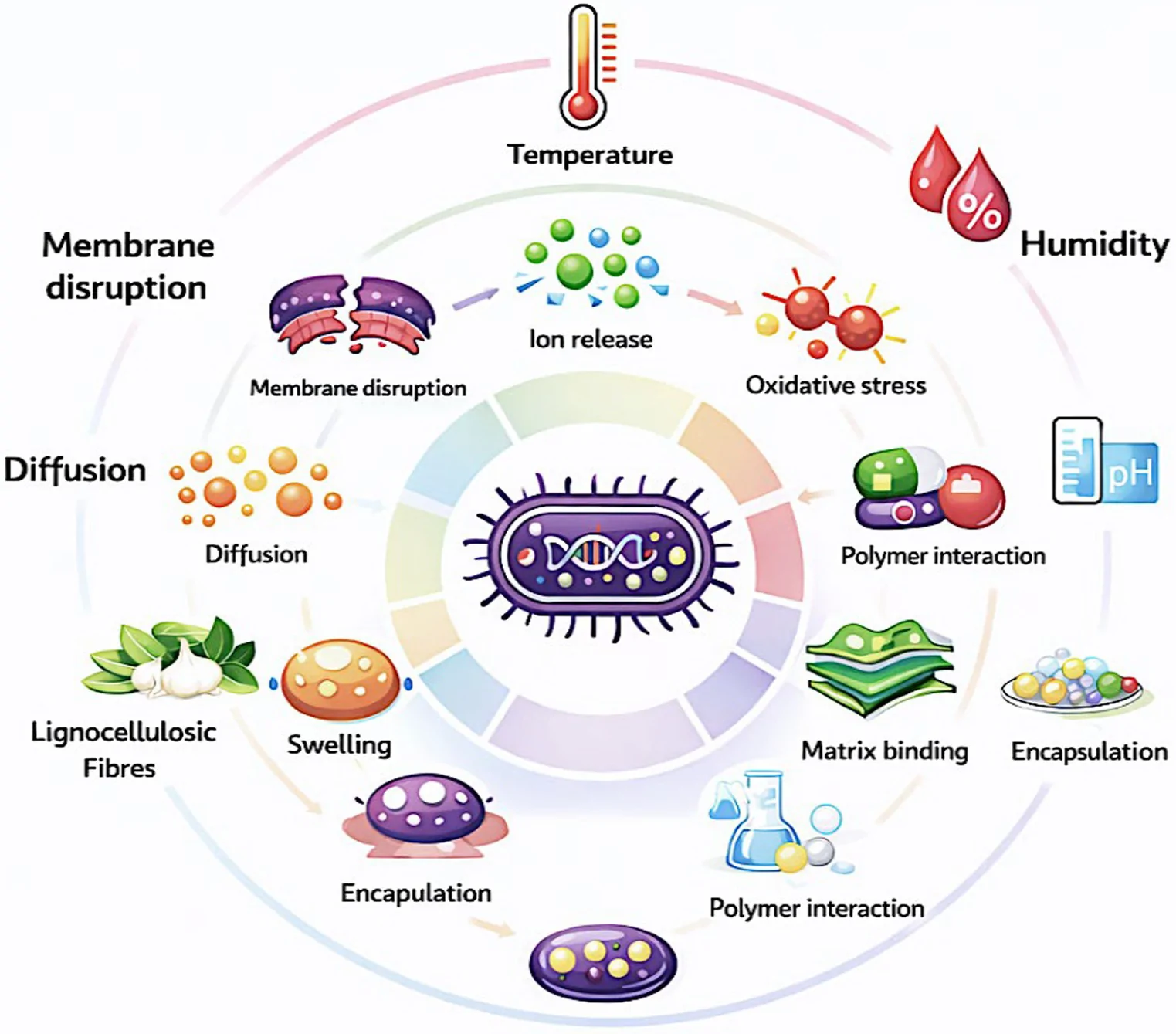
Mechanisms governing antimicrobial activity and controlled-release in bio-packaging materials.

### 6.1 Natural antimicrobial mechanisms

Essential oils, phenolic compounds, bacteriocins, and organic acids inhibit microbial growth through multiple cellular targets and biochemical pathways. Essential oils partition into microbial lipid bilayers, disrupt membrane organization, increase membrane permeability, and promote leakage of intracellular constituents, including ions, amino acids, and ATP (25, 47). Phenolic compounds induce oxidative stress and disrupt enzymatic processes, reducing metabolic activity and microbial viability (13, 77). Nisin and related bacteriocins inhibit microbial growth through interactions with the bacterial cell envelope. Pore formation and disruption of cell-wall biosynthesis contribute to antimicrobial activity, particularly against Gram-positive microorganisms and *Listeria monocytogenes* (75, 78).

Organic acids reduce intracellular pH and disrupt enzyme activity and cellular metabolism, leading to growth inhibition under food-storage conditions (14, 75). Compound stability influences the duration of antimicrobial activity during storage, whereas target susceptibility influences the extent of microbial inhibition (12, 15). Duration of antimicrobial performance depends on compound stability and continued exposure of microbial cells to active agents throughout storage. Essential oils, phenolic compounds, bacteriocins, and organic acids target multiple cellular structures and biochemical pathways, broadening antimicrobial effectiveness in active bio-packaging systems.

### 6.2 Inorganic and nano-enabled antimicrobial materials

ZnO, TiO_2_, and CuO nanoparticles represent the most widely studied inorganic antimicrobial materials for food-packaging applications. Reactive oxygen species (ROS) generated at nanoparticle surfaces damage microbial membranes, proteins, lipids, and nucleic acids, disrupting essential cellular functions and reducing microbial viability (16, 98). ZnO nanoparticles display broad antimicrobial activity because ROS generation occurs alongside Zn^2+^ release. Zn^2+^ ions disrupt membrane integrity and interfere with microbial enzyme systems, leading to suppression of microbial growth (17, 73).

Hybrid ZnO/graphene oxide/montmorillonite coatings further demonstrate the practical antimicrobial potential of nano-enabled packaging systems. Antimicrobial inhibition of approximately 60–74% has been reported against both Gram-positive and Gram-negative microorganisms, highlighting the effectiveness of multifunctional nanocomposite coatings for controlling microbial contamination in food-packaging applications (59). TiO_2_ and CuO nanoparticles contribute antimicrobial functionality primarily through oxidative and surface-mediated mechanisms that impair cellular integrity and metabolic activity (79, 80).

Nanoparticle characteristics strongly influence antimicrobial performance. Particle size and surface properties affect surface reactivity and microbial contact, whereas uniform dispersion promotes consistent antimicrobial functionality throughout packaging materials (10, 20). Light exposure further modifies nanoparticle activity because ultraviolet and visible radiation stimulate ROS generation at photoactive surfaces (79, 80). ROS concentration influences both antimicrobial efficacy and packaging performance. Insufficient ROS generation limits microbial inhibition, whereas excessive ROS production can accelerate oxidation of food constituents (64, 76). Effective antimicrobial performance requires nanoparticle activity sufficient for microbial control while avoiding undesirable effects on food quality and material stability.

### 6.3 Mechanistic pathways of microbial inhibition

Membrane disruption, oxidative stress, ion release, and photodynamic activity represent the principal antimicrobial pathways operating in nano-enabled packaging materials. Electrostatic interactions between antimicrobial compounds and microbial cell surfaces destabilize membrane structure, increase permeability, and promote leakage of intracellular constituents (17, 75). Reactive oxygen species (ROS) damage membrane lipids, proteins, and nucleic acids, leading to loss of cellular function and reduced microbial viability (16, 61). Zn^2+^ ions provide an additional antimicrobial pathway through disruption of membrane potential, enzymatic function, and intracellular biochemical processes (17, 98).

Acidic food environments increase ion availability and influence the extent of antimicrobial activity (17, 20). Photodynamic activity provides a further antimicrobial pathway in selected packaging materials. Light exposure stimulates ROS formation at photoactive surfaces and enhances microbial inhibition beyond direct membrane interactions (79, 80). Membrane damage, oxidative injury, ionic disruption, and photodynamic activity frequently occur simultaneously within antimicrobial packaging systems. Interactions among these mechanisms determine microbial inhibition across multiple cellular targets and contribute to preservation performance during storage (76).

The relative contribution of membrane disruption, oxidative injury, ionic stress, and photoactivated processes varies according to nanoparticle composition, dispersion state, environmental conditions, and microbial susceptibility. Multifunctional nanocomposite systems therefore frequently achieve antimicrobial activity through simultaneous action on several cellular targets rather than through a single dominant inhibitory pathway (16, 59, 76).

### 6.4 Environmental and food-system influences

Storage conditions and food composition influence antimicrobial performance in active-packaging materials. Temperature affects molecular mobility and chemical reactivity, whereas moisture influences transport behavior within polymer matrices (46, 81). Variations in these environmental factors alter antimicrobial availability during storage. Food composition also influences antimicrobial performance through interactions with active compounds. Lipid-rich foods reduce the availability of hydrophobic antimicrobials at microbial interfaces, whereas proteins bind active molecules and modify antimicrobial functionality (25, 75).

Delivery route further influences antimicrobial efficacy. Vapor-phase antimicrobial systems depend on headspace concentration, volatility, gas transport, and package geometry, whereas direct-contact systems rely primarily on compound availability at food surfaces. Differences in transport behavior therefore influence preservation performance in different food categories and storage environments (74, 82).

Food acidity influences the behavior of metal-based antimicrobial systems. Acidic conditions increase Zn^2+^ availability and strengthen antimicrobial performance in susceptible food products (16, 17). Differences among meat, seafood, dairy products, and fresh produce create distinct requirements for antimicrobial packaging design because food composition governs antimicrobial exposure during storage (28, 53). Interactions among antimicrobial agents, packaging materials, food matrices, and storage environments determine preservation outcomes across different food systems. Effective packaging design requires alignment of antimicrobial functionality with the physicochemical characteristics and storage conditions of specific food products (10, 20).

## 7 Nanostructural design and functional performance

Nano-enabled packaging materials combine multiple functional attributes within a single material platform. Polymer composition, nanostructure organization, and active components collectively influence barrier protection, mechanical stability, antimicrobial functionality, and ultraviolet shielding (28, 53). Barrier protection limits environmental exposure, mechanical stability preserves package integrity, antimicrobial functionality suppresses microbial growth, and ultraviolet shielding reduces photo-oxidative deterioration during storage (47, 83). Integration of these functions within a single material system supports food protection, quality retention, shelf-life extension, and more efficient utilization of sustainable packaging materials across diverse food applications (28, 53). Figure 7 highlights relationships between nanostructural organization and key functional attributes of nano-enabled bio-packaging materials.

**Figure 7:**
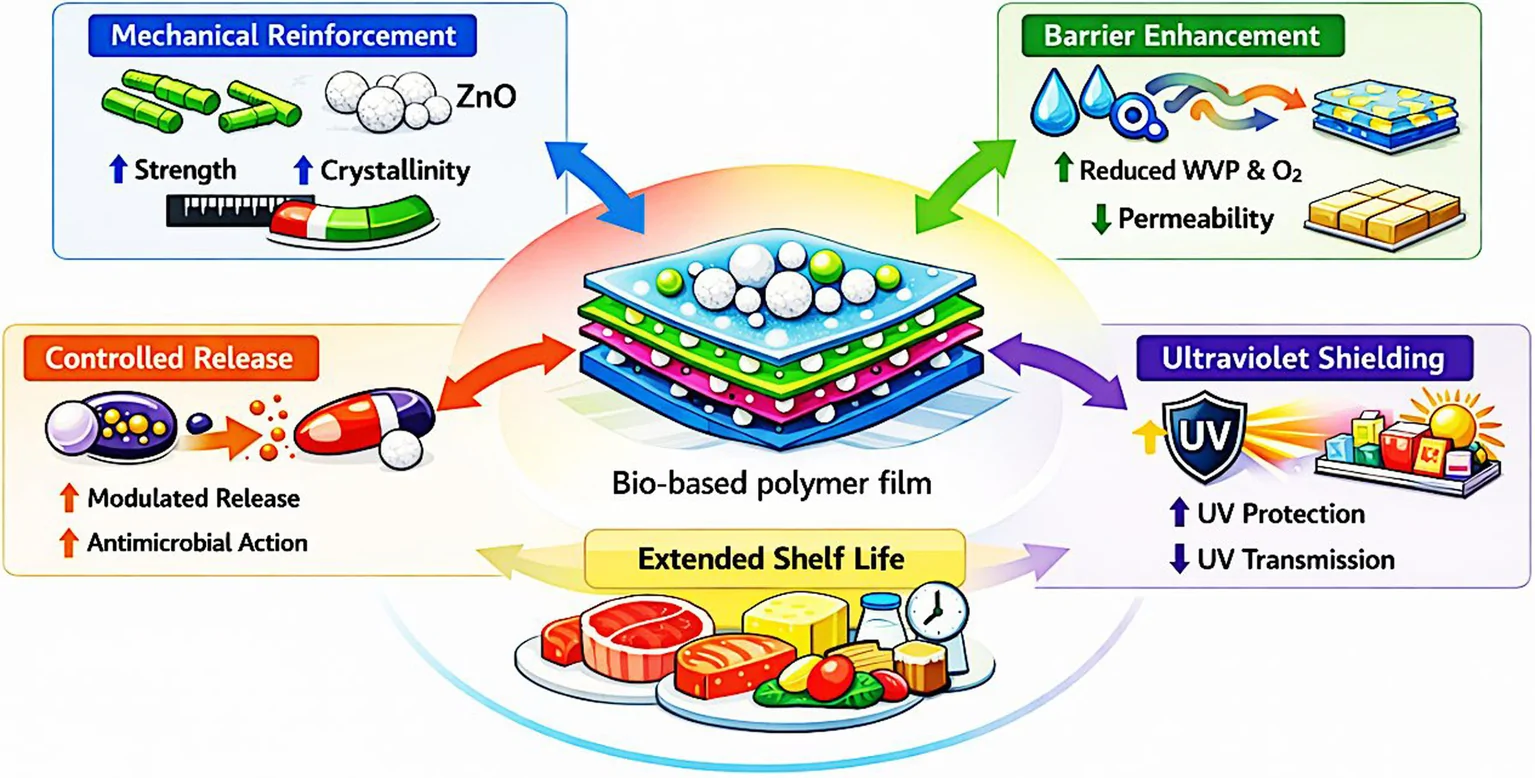
Relationships between nanostructural organization and key functional attributes of nano-enabled bio-packaging materials.

### 7.1 Barrier properties and molecular mobility control

Oxygen, water vapor, and volatile compounds move through packaging materials according to the structural characteristics of the polymer matrix. Dense molecular packing and strong intermolecular interactions reduce permeability in bio-based packaging materials (9, 10). Nanostructure incorporation modifies transport pathways within the matrix and influences barrier performance (10, 19). Polysaccharide nanocrystals and nanofibrils improve barrier properties through formation of compact and highly organized matrix structures (31, 84). Matrix uniformity and nanostructure dispersion further contribute to barrier stability (19).

Chitosan-clay coating systems further demonstrate how nanostructural modification can strengthen barrier performance through reduced oxygen permeability and increased diffusion-path complexity, highlighting the importance of interfacial organization in molecular-mobility control (85). ZnO nanostructures enhance barrier performance in PHBHHx matrices through modification of matrix organization and nanostructure-polymer interactions. Polymer composition, nanostructure distribution, and matrix organization govern resistance to oxygen, moisture, and volatile-compound transmission, influencing preservation performance during storage (2).

### 7.2 Crystallinity, free volume, and diffusion regulation

Crystalline and amorphous regions influence molecular transport through different structural environments within bio-packaging materials. Ordered crystalline domains restrict molecular movement, whereas amorphous regions provide greater opportunities for diffusion of gasses, moisture, and other small molecules (62, 86). The balance between these regions determines transport behavior throughout the matrix. Higher crystallinity reduces free-volume availability and limits polymer-chain motion, thereby restricting diffusion through packaging materials (10, 86).

PHBHHx provides a balance between structural organization and material flexibility for food-packaging applications (42). ZnO nanostructures influence crystallinity within PHBHHx matrices and modify diffusion behavior through changes in polymer organization. Crystallinity and free-volume distribution regulate diffusion behavior, barrier functionality, and bioactive release in nano-enabled packaging materials (2).

### 7.3 Mechanical reinforcement and structural stability

Mechanical stability determines the ability of packaging materials to withstand processing, transportation, storage, and handling. Nanostructures reinforce polymer matrices through interfacial interactions that strengthen stress transfer and matrix cohesion (18, 40). Cellulose nanocrystals, cellulose nanofibrils, and chitin-derived nanostructures increase tensile strength and stiffness through high aspect ratios and extensive hydrogen-bonding networks (18, 50).

Chitosan nanoparticles provide additional reinforcement through electrostatic interactions and hydrogen bonding within composite materials (34, 72). ZnO nanostructures increase stiffness and structural stability in PHBHHx matrices while maintaining material flexibility under storage conditions (2, 48). Uniform nanostructure dispersion limits formation of cracks, microvoids, and other structural defects that compromise material integrity. Mechanical reinforcement improves durability and helps maintain packaging functionality during storage, transportation, and handling (19).

### 7.4 Optical properties, ultraviolet shielding, and photostability

Ultraviolet radiation accelerates oxidative deterioration in foods and packaging materials during storage. ZnO and TiO_2_ nanoparticles absorb ultraviolet radiation and reduce transmission through biodegradable polymer films because of their optical properties and wide bandgap energies (17, 73). Reduced ultraviolet exposure improves the stability of light-sensitive food constituents, pigments, vitamins, and antimicrobial compounds embedded within packaging matrices (79, 80, 87). Nanoparticle concentration and dispersion influence optical performance because light absorption, scattering, and transmission vary according to particle loading and distribution within the polymer matrix (17, 87). Uniform nanostructure dispersion generally improves ultraviolet attenuation and photostability relative to agglomerated systems (87).

ZnO-coated PET and PHBHHx materials further demonstrate the contribution of nanoparticle dispersion to ultraviolet shielding, with ZnO nanostructures providing effective attenuation of ultraviolet transmission while maintaining the functional properties of packaging substrates (88). Ultraviolet shielding reduces photo-oxidative deterioration and improves stability of both packaged foods and active packaging components during storage (2).

### 7.5 Multifunctional performance

Nano-enabled packaging materials combine multiple functional attributes within a single material platform. Polymer composition, nanostructure organization, and active components collectively influence barrier protection, mechanical stability, antimicrobial functionality, and ultraviolet shielding (28, 53). Barrier protection limits environmental exposure, mechanical stability preserves package integrity, antimicrobial functionality suppresses microbial growth, and ultraviolet shielding reduces photo-oxidative deterioration during storage (47, 83). Integration of these functions within a single material system enables simultaneous food protection, quality preservation, and shelf-life extension across diverse food applications (28, 53). Interactions among nanostructure organization, material composition, and active functionality ultimately govern overall packaging performance in next-generation bio-packaging systems.

## 8 Intelligent and smart packaging systems in bio-based materials

Intelligent and smart packaging systems extend the role of bio-based packaging beyond product protection by providing information on food quality, storage conditions, and spoilage status. Indicator and sensing platforms detect physicochemical and microbiological changes associated with food deterioration and generate measurable responses during storage (29, 58). Signal generation depends on interactions between sensing elements and the surrounding food environment. Changes in pH, gas composition, temperature, and microbial activity alter the properties of indicator compounds and produce responses associated with product status (28, 89). pH-responsive indicators, gas-sensitive materials, biosensors, and temperature-responsive systems provide information related to freshness and spoilage progression in packaged foods (29, 89). Figure 8 displays the principal intelligent sensing approaches used in bio-based packaging materials and highlights relationships between nanomaterials, antimicrobial functionality, controlled release, and spoilage monitoring.

**Figure 8:**
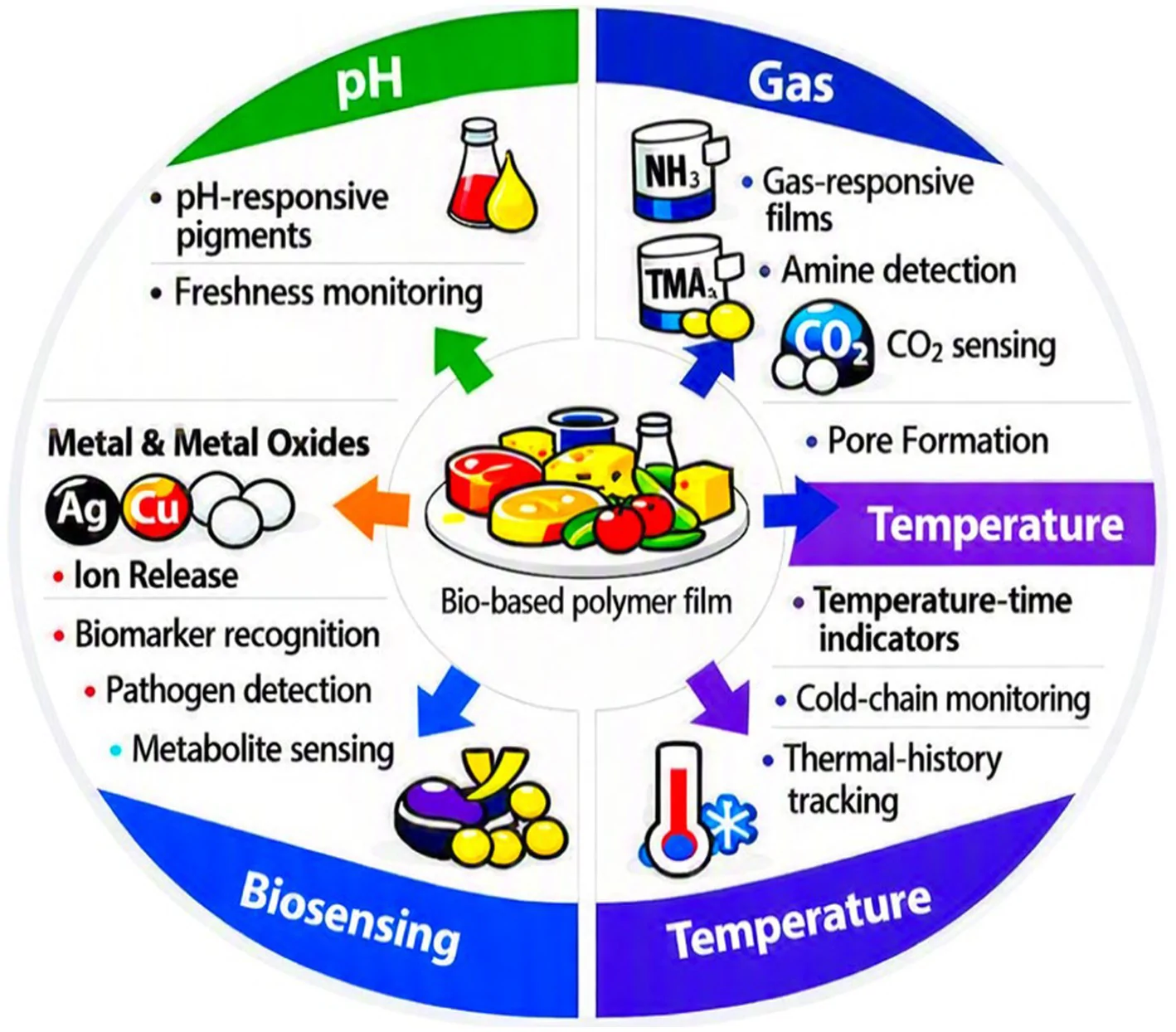
Intelligent sensing platforms and antimicrobial functionalities in bio-based packaging materials.

### 8.1 pH-responsive indicators for freshness monitoring

pH-responsive indicators provide a visual response to biochemical changes associated with food spoilage. Anthocyanins, betalains, curcumin, and chlorophyll undergo color changes across different pH ranges and permit rapid assessment of freshness during storage (29, 89). Changes in food chemistry alter pigment structure and produce visible color transitions that correspond to product status. Anthocyanins display red, purple, blue, and green coloration according to pH conditions and respond to amine accumulation in meat and seafood products. Beetroot-derived indicator films exhibit measurable color changes during refrigerated storage that closely track spoilage progression (58).

Encapsulation systems and polymer-pigment interactions improve pigment stability by increasing retention and reducing sensitivity to environmental fluctuations (13, 37). Improved pigment stability enhances signal reliability and supports accurate freshness assessment throughout storage. Integration of natural pigments within biodegradable matrices enables low-cost freshness monitoring while maintaining compatibility with sustainable packaging systems (28, 58).

### 8.2 Gas-sensing indicators and volatile detection

Gas-sensing technologies detect volatile compounds released during food deterioration and provide early indications of spoilage. Amines, carbon dioxide, and hydrogen sulfide serve as markers of quality loss in meat, seafood, and other perishable foods (28, 29). Volatile compounds accumulating within package headspace provide early indicators of deterioration and can be monitored without direct contact between sensing elements and food products. Headspace-responsive systems therefore complement non-contact preservation approaches based on volatile antimicrobial compounds (74, 82).

Colorimetric films containing natural pigments, metal salts, or nanostructured components respond to these compounds and generate visible signals during storage. ZnO and TiO_2_ nanostructures interact with volatile molecules through highly reactive surface sites and influence sensing performance (16, 65). Sensor performance depends on both the sensing element and the surrounding packaging matrix, while environmental exposure influences signal accuracy and operational durability during storage. Sensing chemistry and material design determine sensitivity, signal stability, and reliability of volatile-compound detection during storage (28, 89).

### 8.3 Temperature–time indicators

Temperature–time indicators record cumulative thermal exposure during storage and distribution. Irreversible color changes occur when predefined temperature thresholds are exceeded, providing a visual record of temperature history (28, 29). Cold-chain monitoring commonly employs these systems because temperature abuse accelerates microbial proliferation and product deterioration in chilled foods. Curcumin- and betalain-based biodegradable indicators exhibit color transitions when storage temperatures exceed recommended limits, allowing identification of temperature excursions (58, 90).

Nanostructures improve indicator reliability by reducing photodegradation and preserving color contrast under fluctuating storage conditions (65, 79). The integration of temperature–time indicators with active packaging systems improves cold-chain monitoring and supports quality management throughout storage and distribution (28, 29).

### 8.4 Optical and fluorescence-based sensing technologies

Optical and fluorescence-based sensing technologies detect physicochemical and biological changes through alterations in light absorption, emission, and photoluminescent behavior. Carbon-dot-reinforced films display fluorescence responses across broad pH ranges and facilitate detection of volatile compounds associated with food deterioration (65, 89, 90).

Photoactive pigments and semiconductor nanostructures respond to light exposure through ROS generation, combining sensing functionality with antimicrobial activity within a single material system. Optical performance depends on matrix stability because photodegradation, moisture uptake, and structural deterioration can compromise signal reliability during storage. Matrix stability therefore remains important for accurate detection and long-term sensor functionality in intelligent packaging systems (79, 80). Optical sensing platforms illustrate how nanomaterials can simultaneously support signal generation, antimicrobial activity, and material protection within integrated intelligent-packaging systems (65, 80).

### 8.5 Multifunctional packaging systems

Food-quality monitoring and antimicrobial protection operate within the same packaging materials. Encapsulation systems and multilayer architectures separate sensing elements from antimicrobial agents. Spatial separation reduces functional interference and improves signal reliability (13, 28, 47, 63). Biopolymer matrices require suitable mechanical stability, permeability characteristics, and resistance to photodegradation for simultaneous sensing and preservation functions. Matrix properties influence the retention of sensing elements and antimicrobial compounds during storage. Intelligent indicators detect physicochemical changes associated with spoilage progression, whereas antimicrobial components reduce microbial growth within the same package environment. Shelf-life management and food-quality assurance benefit from real-time monitoring combined with antimicrobial protection during storage and distribution (13, 28).

Hybrid ZnO/graphene oxide/montmorillonite coatings further demonstrate how nanostructured materials can simultaneously provide antimicrobial functionality, barrier enhancement, and structural reinforcement, illustrating the multifunctional design principles increasingly employed in advanced intelligent-packaging systems (59).

Intelligent packaging systems transform packaging materials from passive barriers into active information platforms capable of monitoring quality, detecting spoilage, and supporting preservation strategies throughout storage and distribution. The integration of sensing technologies with controlled-release antimicrobial systems, nanostructured materials, and sustainable biopolymer matrices provides a foundation for next-generation packaging systems that combine food protection, quality assessment, and environmental responsibility (13, 27, 28).

## 9 Migration behavior, safety, and regulatory considerations

Antimicrobial compounds, nanoparticles, and degradation products may migrate from packaging materials into food systems during storage. Material composition, packaging structure, food characteristics, and storage conditions influence migration behavior (20, 46). Food-contact safety depends on the evaluation of substances transferred from packaging materials to food during intended use (21).

Antimicrobial functionality requires active compounds to remain available at concentrations sufficient for microbial control, whereas excessive transfer into food products may increase consumer exposure and compromise food-contact safety (20, 81). Material selection, packaging design, and exposure conditions influence this balance during storage. Risk assessment of nano-enabled packaging materials considers nanoparticle behavior, degradation products, consumer exposure, and toxicological effects.

Safe-by-design strategies reduce unintended exposure through control of material composition and packaging architecture (21, 64). Regulatory frameworks establish requirements for migration testing, safety assessment, and market approval of food-contact materials. Figure 9 illustrates migration pathways, toxicological considerations, safe-by-design strategies, and regulatory frameworks relevant to nano-enabled bio-packaging materials.

**Figure 9:**
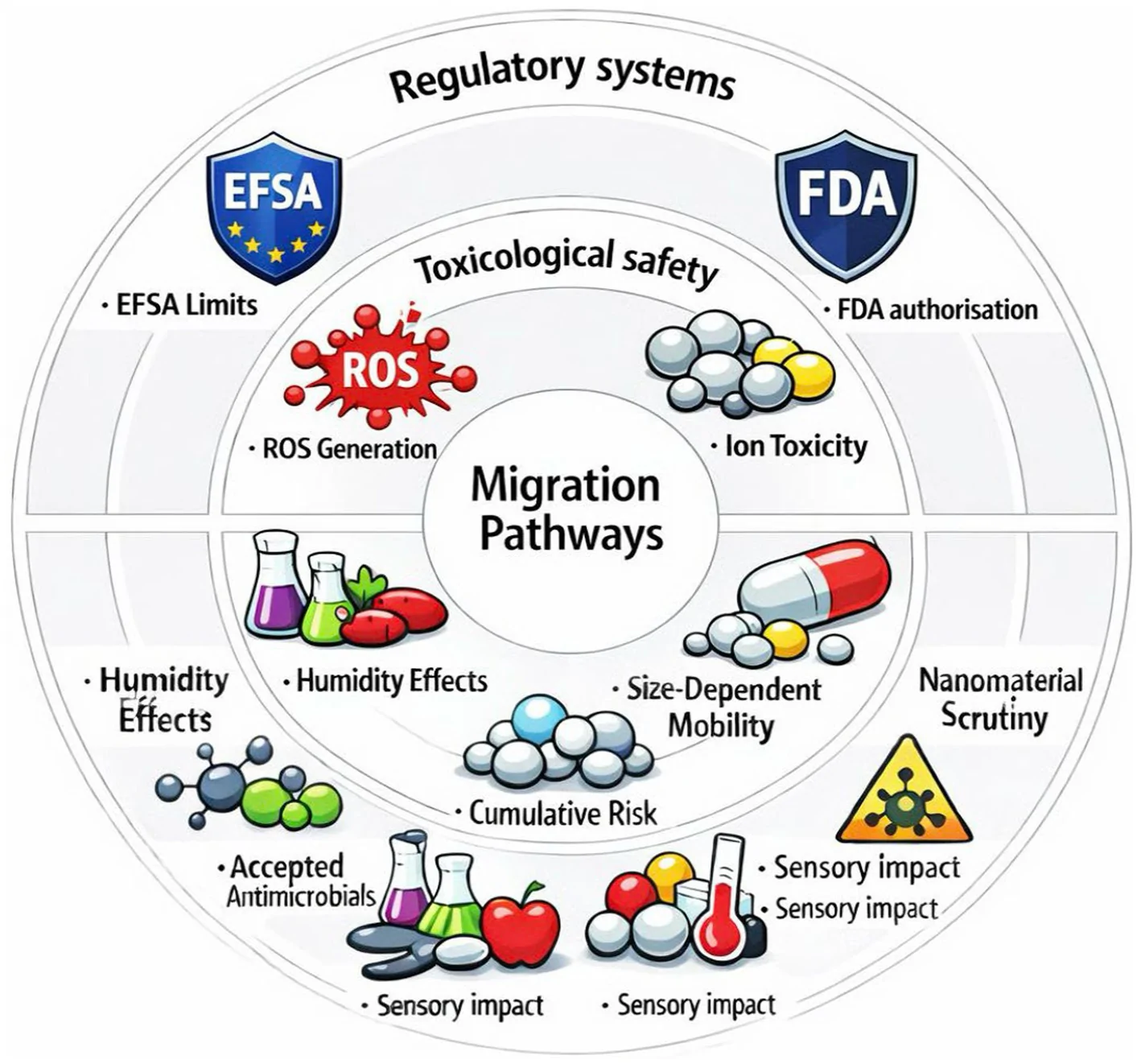
Migration pathways, safety considerations, and regulatory frameworks in nano-enabled bio-packaging materials.

### 9.1 Mechanisms governing migration in bio-packaging materials

Diffusion governs the movement of antimicrobial compounds, nanoparticles, ions, and degradation products from packaging materials into food systems. Polymer morphology, crystallinity, free-volume distribution, matrix hydrophobicity, and polymer-additive interactions influence migration behavior (20, 46). Temperature, storage duration, food composition, and water activity modify migration behavior within packaging materials (20, 81).

Hydrophilic biopolymer matrices, including starch-, cellulose-, and chitosan-based materials, often exhibit greater migration under humid conditions. Water absorption promotes swelling, modifies matrix organization, and increases movement of incorporated substances within the material (30, 46). Hydrophobic polymer systems exhibit lower water uptake and reduced mobility of incorporated compounds under comparable storage conditions (42).

Nanoparticle characteristics also influence migration behavior. Particle size, agglomeration state, surface chemistry, and distribution within polymer matrices affect retention and potential release into food systems (17, 20). Food composition further modifies migration profiles. Migration behavior therefore reflects interactions among material properties, food composition, and storage conditions rather than the influence of a single factor (20, 21).

### 9.2 Migration of antimicrobial compounds and nanomaterials

Migration of active substances depends on the physicochemical properties of both the migrant and the food-contact environment. Molecular size, polarity, volatility, and matrix affinity influence the migration of organic antimicrobial compounds (75, 81). Hydrophilic compounds generally display greater migration in moisture-rich foods, whereas hydrophobic compounds exhibit stronger retention within polymer matrices but may partition into lipid-rich food systems (75).

Metal and metal-oxide nanomaterials exhibit distinct migration behavior because exposure may occur through nanoparticle transfer and ion release. ZnO nanostructures release Zn^2+^ ions under food-contact conditions. Nanoparticle concentration, particle dimensions, surface properties, and matrix compatibility influence migration behavior. Reported studies have identified migration values of approximately 43 mg/L for ZnO under severe exposure conditions and values approaching 1,091 mg/L for zinc pyrithione under worst-case scenarios (21).

Polymer-additive interactions, nanofiller distribution, and matrix organization influence retention of active substances within packaging matrices. Structural characteristics regulate mobility of antimicrobial compounds and nanomaterials during storage and food contact (10, 86). Migration assessment therefore provides an essential link between antimicrobial functionality, consumer exposure, and food-contact safety in active and nano-enabled packaging systems (10, 21).

### 9.3 Nano-toxicity and human health considerations

Nanoparticles, released ions, and reactive intermediates may interact with biological systems following migration from food-contact materials. Particle size, surface chemistry, solubility, concentration, and bioavailability influence toxicological responses to nanomaterial exposure (17, 61). Metal and metal-oxide nanomaterials generate ROS and release bioactive ions that interact with cellular membranes, proteins, and nucleic acids (17, 61). Excessive exposure to ZnO and CuO nanomaterials has been associated with oxidative stress, mitochondrial dysfunction, inflammatory responses, and disturbances of cellular metabolism under experimental conditions (17).

Polymer matrices restrict nanoparticle mobility through immobilization of dispersed nanomaterials and reduce opportunities for direct biological interaction (15). Polymer-particle interactions influence nanoparticle retention within packaging materials and regulate potential release into food-contact environments (15, 20). Consumer safety requires effective antimicrobial functionality while limiting exposure to migrated substances. Migration assessment and toxicological evaluation therefore remain integral components of safety assessment for nano-enabled food-packaging materials (10, 20).

### 9.4 Regulatory frameworks and compliance requirements

Commercial application of antimicrobial and intelligent packaging materials requires compliance with food-contact regulations established by authorities, including the European Food Safety Authority (EFSA) and the United States Food and Drug Administration (FDA) (65, 91). Regulatory assessment considers intended-use conditions, migration behavior, toxicological safety, dietary exposure, and potential effects on food quality (91). Nisin and organic acids possess established safety records and broad regulatory acceptance, whereas engineered nanomaterials require additional evaluation because uncertainties remain regarding long-term exposure, bioaccumulation, and environmental persistence (65).

Migration studies, toxicological testing, and exposure assessment constitute key requirements for nano-enabled packaging materials (21). Pigments, enzymes, sensors, antimicrobial compounds, and nanoscale additives require assessment within the final packaging system before commercial use (21, 28). Multilayer packaging structures reduce direct food contact through confinement of active components within designated layers (21, 63).

Safety evaluation under realistic storage and food-contact conditions remains necessary for commercial implementation. Regulatory approval depends on integration of migration data, exposure assessment, and toxicological evidence to demonstrate food-contact safety under intended conditions of use (21, 28).

### 9.5 Safe-by-design strategies for nano-enabled packaging

Safe-by-design strategies minimize consumer exposure while preserving packaging functionality. Material selection, nanostructure engineering, and packaging design support control of nanoparticle mobility and antimicrobial release. Encapsulation technologies confine antimicrobial compounds within protective carrier systems and reduce unintended release into food products (14, 15). Surface modification and homogeneous nanofiller distribution increase nanostructure retention within packaging materials (18, 44).

Polymer-particle interactions limit nanoparticle mobility and reduce potential exposure during storage (15). Multilayer packaging structures provide an additional safety strategy. Confinement of active components within designated layers reduces direct food contact while maintaining packaging functionality (28, 63). Safe-by-design strategies seek to maintain antimicrobial performance while reducing unintended migration and consumer exposure, thereby supporting both functionality and regulatory compliance (15, 28).

### 9.6 Balancing migration restriction and antimicrobial delivery

Antimicrobial packaging requires control of microbial growth alongside food-contact safety. Antimicrobial compounds must remain available at concentrations sufficient to suppress microbial growth while limiting transfer into food products (10, 20). Release behavior influences antimicrobial availability throughout storage. Rapid release shortens antimicrobial functionality, whereas excessive retention within packaging materials reduces preservation efficacy (10, 13).

Material composition and structural organization govern both antimicrobial delivery and migration behavior. Antimicrobial availability influences preservation efficacy, whereas consumer exposure influences food-contact safety. Successful packaging design therefore depends on balancing sustained antimicrobial functionality with controlled migration under intended conditions of use. Evaluation of antimicrobial efficacy, migration behavior, and food-contact safety requirements remains essential for commercial implementation of active-packaging systems because preservation performance and consumer protection depend on maintaining an appropriate balance between antimicrobial availability and controlled migration under intended conditions of use (20, 21).

## 10 Applications and sustainability of next-generation bio-packaging systems

Food preservation, shelf-life extension, and food-contact safety determine the practical application of antimicrobial and intelligent bio-based packaging materials. Bio-based packaging systems reduce microbial contamination, delay spoilage, and maintain food quality across diverse food categories (25, 36). Intelligent monitoring technologies provide information on freshness, spoilage progression, and storage conditions during distribution and storage (28). Product composition, storage conditions, and spoilage characteristics influence packaging requirements and preservation performance across different food systems (47).

Environmental sustainability influences packaging development and material selection. Renewable feedstocks, biodegradation behavior, resource efficiency, and compatibility with circular-economy strategies determine environmental performance throughout the material life cycle (23, 56). Bio-based packaging systems utilize renewable feedstocks and biodegradable end-of-life pathways that reduce dependence on petroleum-derived materials. Figure 10 showcases relationships among biopolymer classes, biodegradation pathways, environmental performance, and sustainable end-of-life options relevant to next-generation bio-packaging systems.

**Figure 10:**
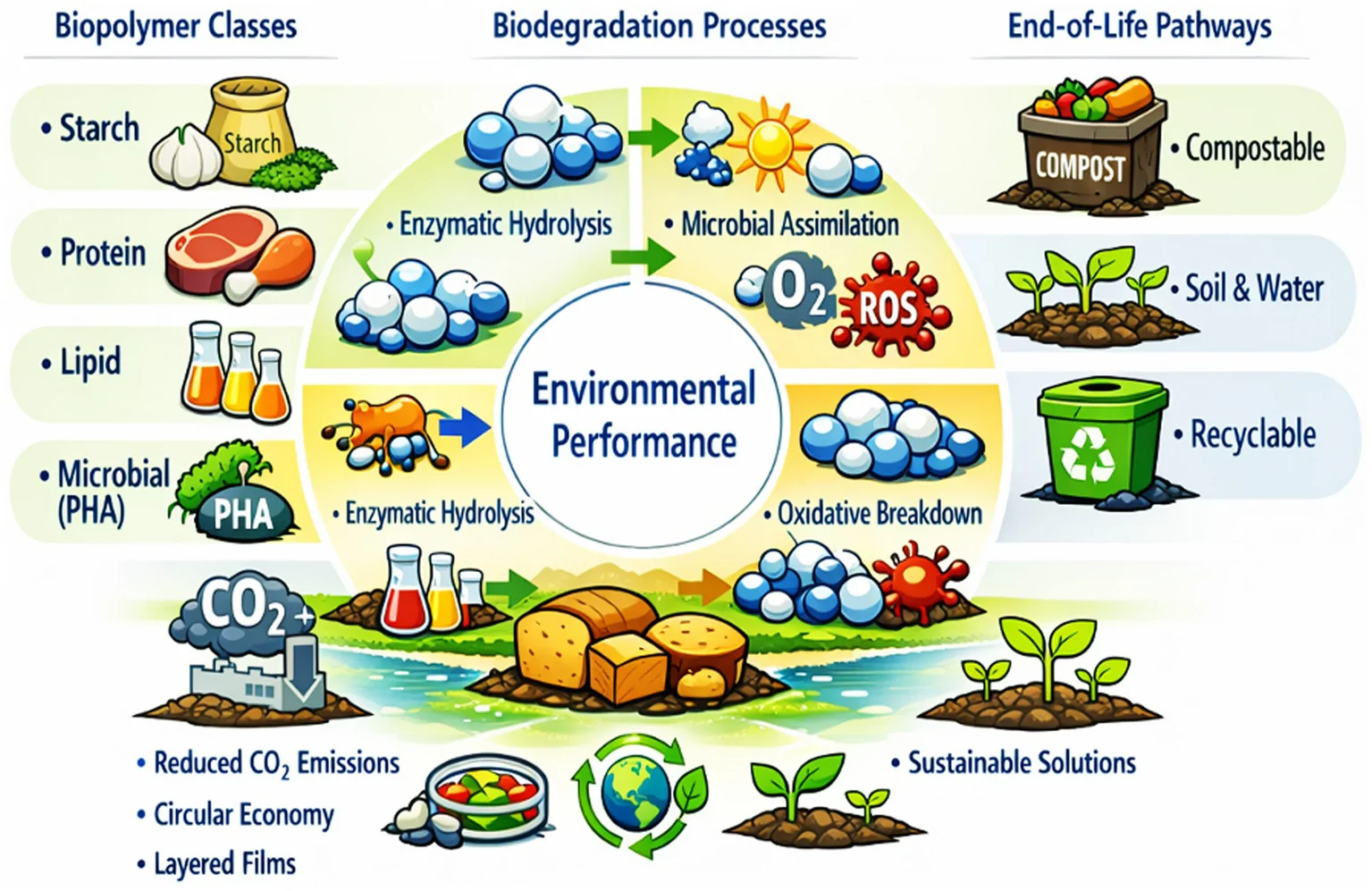
Environmental performance and end-of-life pathways of bio-based packaging materials.

### 10.1 Applications in real food systems

Meat and poultry products represent demanding packaging applications because high water activity, abundant nutrients, and diverse microbial populations accelerate spoilage. Essential-oil-containing films, bacteriocin-based coatings, and ZnO-reinforced materials inhibit the growth of *Escherichia coli*, *Staphylococcus aureus*, and *Listeria monocytogenes* while reducing oxidative deterioration during storage (25, 75). High-barrier multilayer packaging systems with oxygen transmission rates as low as 3.95 cm^3^/m^2^/day reduced microbial growth, decreased the relative abundance of *Pseudomonas*, and extended the shelf life of refrigerated yak meat to 20 days, demonstrating the practical importance of barrier performance in food-preservation applications (97).

Seafood products present additional challenges because microbial growth and spoilage reactions progress rapidly under chilled conditions. pH-responsive indicators detect volatile amines generated during deterioration, whereas antimicrobial films suppress microbial proliferation during storage (29, 58). Dairy products utilize antimicrobial coatings and reinforced packaging materials that reduce microbial growth and oxidative deterioration. Ultraviolet-shielding materials protect oxidation-sensitive food components during illuminated storage (16, 79).

Fresh fruits and vegetables require packaging systems that manage respiration, moisture loss, and microbial contamination after harvest. Gas-sensitive indicators monitor atmospheric changes within packages, whereas antimicrobial materials reduce microbial contamination during storage and distribution (31, 84, 93). Bakery and cereal products remain susceptible to mold growth and moisture fluctuations during storage. Antimicrobial barrier materials suppress fungal development, stabilize moisture conditions, and preserve product quality throughout prolonged storage periods (47, 80).

Ready-to-eat foods require preservation and quality monitoring under variable storage and retail conditions. Multifunctional packaging systems combine intelligent sensing with antimicrobial protection within the same packaging platform (28, 53). Multilayer packaging architectures maintain signal reliability and preservation performance through spatial separation of sensing and antimicrobial domains (63). Diverse food matrices impose distinct preservation challenges, requiring packaging systems that combine barrier protection, antimicrobial functionality, intelligent monitoring, and food-contact safety according to product-specific storage requirements (28, 53).

### 10.2 Antimicrobial release in food systems

Antimicrobial efficacy depends on sufficient concentrations of active compounds at food surfaces throughout storage. Food composition, storage conditions, and antimicrobial properties influence active-compound availability within food systems (75, 81). Hydrophilic compounds generally exhibit greater mobility in moisture-rich foods, whereas hydrophobic compounds often display stronger matrix retention and greater affinity for lipid-containing products (75, 81).

Temperature and humidity modify active-compound availability through their effects on transport processes within packaging materials and food systems (13, 81). Release behavior determines antimicrobial availability during storage. Rapid release shortens antimicrobial functionality, whereas excessive retention within packaging materials reduces preservation efficacy. Packaging design therefore governs antimicrobial availability throughout storage and influences preservation efficacy across different food systems (10, 13).

### 10.3 Migration behavior in food applications

Migration behavior under food-storage conditions depends on interactions among packaging materials, food composition, and storage environments. Food chemistry, moisture content, temperature, and storage duration influence transfer of active compounds and nanoparticles from packaging materials into food systems (20, 46).

Food composition modifies migration behavior through interactions with active compounds and nanomaterials. Lipid-rich foods promote partitioning of hydrophobic antimicrobial compounds, whereas acidic environments increase dissolution and ion release from metal-oxide nanomaterials (17, 21). Food-contact safety requires migration levels within regulatory limits while maintaining antimicrobial functionality. Migration assessment under intended storage and use conditions provides an essential basis for balancing antimicrobial functionality with food-contact safety requirements (20, 21).

### 10.4 Environmental sustainability and biodegradation

Biodegradability supports circular-economy objectives through reduced dependence on conventional petroleum-derived plastics (8, 42). Material composition, environmental conditions, and microbial activity influence degradation behavior after disposal (41, 42). Starch-based materials often degrade rapidly under composting conditions because water absorption increases accessibility to microbial and enzymatic attack (41). PHAs undergo biodegradation through enzymatic depolymerisation of polyester chains in soil, compost, freshwater, and marine environments (42, 44).

Temperature, moisture availability, and microbial abundance influence degradation rates (46, 81). Renewable feedstocks, biodegradability, and compatibility with circular-material strategies reduce environmental persistence and strengthen the sustainability profile of bio-based packaging systems throughout their life cycle (8, 56).

### 10.5 Circularity, life-cycle assessment, and environmental impact

Life-cycle assessment evaluates environmental performance across the entire packaging life cycle, from feedstock production to end-of-life management. Renewable biomass and waste-derived feedstocks reduce dependence on fossil resources and may lower environmental burdens relative to conventional plastics (23, 60). Environmental performance depends on biodegradation behavior, resource efficiency, and feedstock origin. Material composition, environmental conditions, and microbial activity influence degradation pathways and end-of-life outcomes for bio-based packaging materials (8, 42).

Antimicrobial and intelligent-packaging functions may also influence sustainability outcomes. Natural antimicrobial compounds generally exhibit compatibility with biodegradation processes, whereas nanomaterial selection and additive loading require careful consideration during material design and disposal (17, 47). Circular-economy performance depends on responsible feedstock selection, efficient resource utilization, appropriate end-of-life management, and comprehensive life-cycle evaluation. Integration of renewable feedstocks, biodegradable materials, intelligent functionality, and responsible material design supports development of packaging systems with improved environmental performance and reduced dependence on fossil-derived resources (53, 56).

## 11 Challenges, limitations, and future directions

Laboratory performance frequently differs from performance under commercial storage, transportation, and food-contact conditions. Environmental variation and food-matrix interactions influence antimicrobial efficacy, barrier properties, sensing reliability, and material stability (8, 30, 81). Variability in polymer composition, nanostructure dispersion, and manufacturing processes influences reproducibility during industrial production (64).

Preservation performance, food-contact safety, environmental sustainability, regulatory compliance, and economic viability must operate within the same packaging system. Antimicrobial delivery, migration control, biodegradation, and intelligent-packaging functions must remain effective across diverse food applications (28, 53). Current research explores advanced biopolymers, safe-by-design nanocomposites, controlled-release technologies, green manufacturing approaches, and circular packaging systems to address these limitations (53, 56). Figure 11 consolidates major challenges associated with bio-based packaging materials and highlights future directions for improving material performance, sustainability, manufacturing, and circularity.

**Figure 11:**
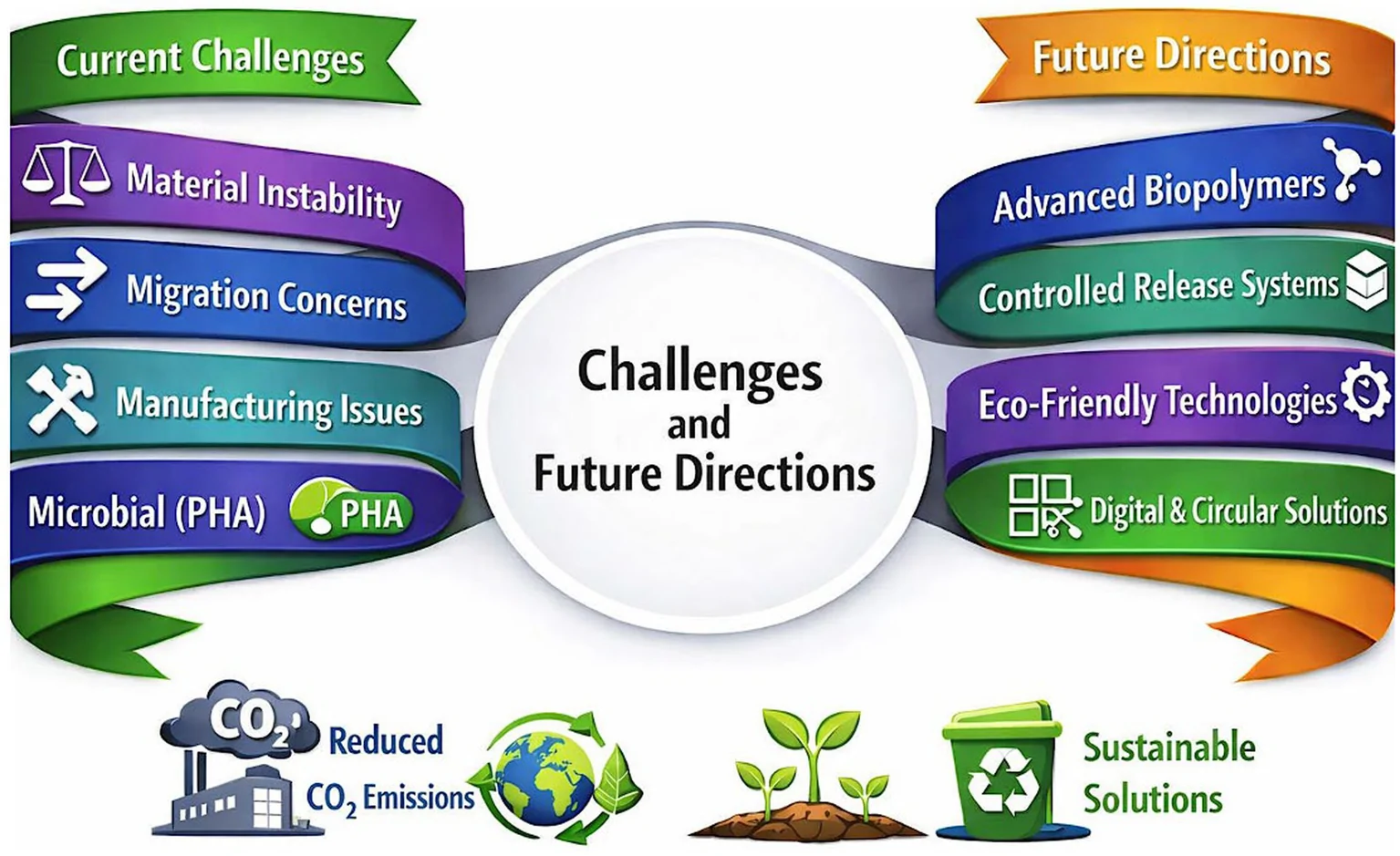
Current challenges and future development pathways for antimicrobial bio-based packaging materials.

### 11.1 Material performance and stability constraints

Moisture absorption, brittleness, thermal instability, and mechanical deterioration limit the performance of many bio-based packaging materials during storage and food-contact applications. Barrier properties, structural integrity, and antimicrobial functionality depend on the stability of the underlying material system (18, 40). Nanostructure incorporation strengthens mechanical properties and barrier stability, although heterogeneous crystallization and weak interfacial interactions may compromise long-term performance within biopolymer matrices (18, 40, 44).

PHBHHx exhibits greater flexibility than PHB and supports applications requiring improved mechanical performance (42). Crystallinity influences molecular transport, barrier properties, and mechanical behavior within bio-based packaging materials. Higher crystallinity restricts molecular transport and improves barrier performance, whereas excessive crystallinity may reduce flexibility under variable storage conditions (48, 83). Polymer morphology influences long-term material stability and functional performance.

### 11.2 Migration restriction and safety considerations

Migration control presents a major challenge in antimicrobial packaging design. Antimicrobial efficacy requires sufficient availability of active compounds, whereas food contact safety requires migration within acceptable exposure limits (10, 20). Particle characteristics, additive concentration, food composition, pH, temperature, and storage duration influence migration behavior within food packaging systems (10, 17, 20). Hydrophilic biopolymer matrices often exhibit greater migration under humid conditions because water absorption modifies matrix organization and increases mobility of incorporated substances (30, 46).

Nanoparticle distribution, matrix organization, and polymer-particle interactions influence retention of active components within packaging materials (20). Safe-by-design strategies reduce unintended exposure through material selection, polymer-particle interactions, and retention of active compounds within the packaging matrix. Food contact applications require antimicrobial activity alongside migration within acceptable exposure limits (13).

### 11.3 Manufacturing and scalability barriers

Commercial production of bio-based packaging materials depends on consistent raw-material quality, process control, and manufacturing reproducibility. Agricultural feedstocks and microbially derived polymers differ in composition and molecular characteristics, producing variation in mechanical properties, barrier behavior, and antimicrobial functionality (54, 56). Processing methods influence nanofiller distribution and batch-to-batch consistency within packaging systems. Nanoparticle agglomeration generates structural heterogeneity and reduces product uniformity during large-scale production (52, 65, 86).

PHBHHx-based materials exhibit greater processability than several conventional PHA systems (42, 48). Industrial-scale manufacture requires reliable processing conditions, homogeneous nanofiller distribution, and consistent product quality. Production costs frequently exceed those of petroleum-derived plastics. Microbial fermentation technologies, bioprocess optimisation, and utilization of waste-derived feedstocks may improve commercial competitiveness and support wider industrial adoption (41, 57).

### 11.4 Regulatory frameworks and consumer acceptance

Commercial application of antimicrobial and intelligent packaging materials requires regulatory approval. Migration behavior, toxicological safety, intended-use conditions, and dietary exposure constitute major components of regulatory assessment (65, 91). Natural antimicrobial compounds often possess established safety records, whereas nanomaterials require additional evaluation because uncertainties remain regarding long-term exposure, transformation, and bioaccumulation (65). Bio-based plastics and their nanocomposites introduce additional regulatory considerations because nanoscale reinforcements can modify migration behavior, surface reactivity, and degradation pathways, requiring assessment under realistic food-contact conditions (92).

Intelligent packaging systems introduce further regulatory requirements. Pigments, enzymes, sensors, nanostructures, and antimicrobial agents require assessment within complete packaging systems. Active and intelligent biobased packaging systems require additional regulatory evaluation because performance assessment must consider antimicrobial functionality, sensing reliability, migration behavior, food-contact safety, long-term material stability, and interactions between antimicrobial and sensing components within the complete packaging architecture (28, 76, 92). Multilayer architectures reduce direct food contact through confinement of active components within designated packaging layers (21, 28, 63).

Consumer acceptance influences the adoption of advanced packaging materials. Public perception is influenced by confidence in food safety, nanoparticle risk communication, sustainability claims, intelligent monitoring functions, and transparency regarding packaging performance (76, 82). Food safety, biodegradability, environmental performance, and label transparency influence public perception. Commercial implementation of bio-based nanocomposite packaging depends on regulatory evaluation of nanomaterial migration, toxicological safety, intelligent sensing components, environmental sustainability, and consumer acceptance alongside packaging performance (21, 92). Clear communication of safety, functionality, and sustainability characteristics supports confidence in bio-based packaging technologies (36, 47, 53).

### 11.5 Future research and innovation pathways

Multifunctional packaging systems require integration of antimicrobial activity, intelligent monitoring, controlled release, food-contact safety, biodegradability, environmental sustainability, and real-time sensing capabilities within a single material platform (76, 82). Different food matrices and storage conditions impose competing material-design requirements that influence the development of integrated packaging systems (28, 53).

Fresh fruits and vegetables require packaging systems that manage respiration, moisture loss, and microbial contamination after harvest. Gas-sensitive indicators monitor atmospheric changes within packages, whereas antimicrobial materials reduce microbial contamination during storage and distribution (31, 84, 93). Plant-mediated nanomaterial production, bio-inspired manufacturing approaches, low-energy fabrication processes, and utilization of renewable feedstocks support reduced environmental burdens and improved resource efficiency within packaging production systems (36, 56, 73).

Nanostructure engineering influences functional integration, migration control, material stability, packaging performance, and controlled antimicrobial delivery through contact-active and release-based mechanisms (18, 82). Surface functionalisation and hybrid nanostructure design improve compatibility between active and intelligent packaging components and regulate antimicrobial release, sensing performance, and physicochemical stability. Multilayer packaging architectures separate sensing and antimicrobial components within the same packaging system and reduce direct food exposure to active nanomaterials (18, 44, 63, 86).

Predictive modeling supports optimisation of antimicrobial release, nanoparticle migration, degradation behavior, and environmental fate. Machine-learning and computational approaches support formulation screening and material selection for specific packaging applications. Commercial deployment of nano-enabled bio-packaging materials depends on reliable manufacturing processes, predictive understanding of release and migration behavior, and robust safety-assessment frameworks. Internationally harmonized testing methodologies, standardized migration and toxicity protocols, performance validation in real food systems, and alignment among food-contact regulations support regulatory approval and commercial adoption (53, 64, 82).

## 12 Conclusion

This review demonstrates that material architecture, nanostructure engineering, molecular transport, and controlled-release behavior collectively govern bioactive delivery, migration characteristics, barrier performance, and food-contact safety in next-generation bio-packaging systems. Bio-based polymers and nano-enabled materials provide versatile platforms for integrating antimicrobial protection, antioxidant activity, intelligent sensing, and sustainability within multifunctional packaging technologies. Crystallinity, matrix organization, nanostructure dispersion, carrier design, and release mechanisms influence the performance, stability, and preservation efficacy of these systems across diverse food applications. Active packaging technologies suppress microbial spoilage and oxidative deterioration, whereas intelligent packaging systems enable real-time monitoring of freshness, spoilage progression, and storage conditions. The integration of preservation and monitoring functions supports improved food quality, extended shelf life, and more effective management of food products throughout storage and distribution. Biodegradability, renewable feedstocks, resource valorisation, and compatibility with circular-bioeconomy principles further strengthen the sustainability potential of advanced bio-packaging materials. Commercial deployment of nano-enabled bio-packaging systems requires scalable manufacturing processes, predictive understanding of release and migration behavior, robust safety-assessment frameworks, standardized performance evaluation, and harmonized regulatory requirements. The evidence presented throughout this review indicates that successful packaging design depends on balancing sustained antimicrobial functionality with controlled migration, material safety, and preservation performance under intended conditions of use. Advances in material engineering, active packaging technologies, intelligent sensing platforms, and sustainable design strategies provide a foundation for the development of safer, more functional, and environmentally responsible food packaging solutions.
